# Interaction of lncRNA MIR100HG with hnRNPA2B1 facilitates m^6^A-dependent stabilization of TCF7L2 mRNA and colorectal cancer progression

**DOI:** 10.1186/s12943-022-01555-3

**Published:** 2022-03-12

**Authors:** Hao Liu, Danxiu Li, Lina Sun, Hongqiang Qin, Ahui Fan, Lingnan Meng, Ramona Graves-Deal, Sarah E. Glass, Jeffrey L. Franklin, Qi Liu, Jing Wang, Timothy J. Yeatman, Hao Guo, Hong Zong, Shuilin Jin, Zhiyu Chen, Ting Deng, Ying Fang, Cunxi Li, John Karijolich, James G. Patton, Xin Wang, Yongzhan Nie, Daiming Fan, Robert J. Coffey, Xiaodi Zhao, Yuanyuan Lu

**Affiliations:** 1 State Key Laboratory of Cancer Biology, National Clinical Research Center for Digestive Diseases, Xijing Hospital of Digestive Diseases, Fourth Military Medical University, 127 West Changle Rd, Xi’an, 710032 Shaanxi China; 2 Department of Gastroenterology, Tangdu Hospital, Fourth Military Medical University, Xi’an, 710038 Shaanxi China; 3 The Affiliated Children’s Hospital of Xi’an Jiaotong University, Xi’an, 710003 China; 4 CAS Key Laboratory of Separation Science for Analytical Chemistry, Dalian Institute of Chemical Physics, Chinese Academy of Sciences, Dalian, 116023 Liaoning China; 5 Departments of Medicine and Cell and Developmental Biology, Vanderbilt University Medical Center, 2213 Garland Ave, Nashville, TN 37232 USA; 6 Department of Biomedical Informatics and Center for Quantitative Sciences, Vanderbilt University Medical Center, Nashville, TN 37232 USA; 7 Departments of Surgery and Molecular Medicine, TGH Cancer Institute and University of South Florida, Tampa, FL 33620 USA; 8 State Key Laboratory of Translational Medicine and Innovative Drug Development, Jiangsu Simcere Diagnostics Co., Ltd., Nanjing, 210042 Jiangsu China; 9 The First Affiliated Hospital of Zhengzhou University, Zhengzhou, 450052 Henan China; 10 Department of Medical Oncology, Fudan University Shanghai Cancer Center, Shanghai, 200032 China; 11 Tianjin Medical University Cancer Institute and Hospital, National Clinical Research Center for Cancer, Tianjin’s Clinical Research Center for Cancer, Key Laboratory of Cancer Prevention and Therapy, Tianjin, 300060 China; 12 Jiaen Genetics Laboratory, Beijing Jiaen Hospital, Beijing, 100191 China; 13 Department of Biochemistry, Vanderbilt University Medical Center, Nashville, TN 37232 USA; 14 Department of Biological Sciences, Vanderbilt University School of Medicine, Nashville, TN 37232 USA

**Keywords:** MIR100HG, hnRNPA2B1, TCF7L2, N6-methyladenosine (m^6^A), Wnt/β-catenin signaling, EMT, Cetuximab resistance, Metastasis, CRC

## Abstract

**Background:**

Epithelial-to-mesenchymal transition (EMT) is a process linked to metastasis and drug resistance with non-coding RNAs (ncRNAs) playing pivotal roles. We previously showed that miR-100 and miR-125b, embedded within the third intron of the ncRNA host gene *MIR100HG*, confer resistance to cetuximab, an anti-epidermal growth factor receptor (EGFR) monoclonal antibody, in colorectal cancer (CRC). However, whether the MIR100HG transcript itself has a role in cetuximab resistance or EMT is unknown.

**Methods:**

The correlation between MIR100HG and EMT was analyzed by curating public CRC data repositories. The biological roles of MIR100HG in EMT, metastasis and cetuximab resistance in CRC were determined both in vitro and in vivo. The expression patterns of MIR100HG, hnRNPA2B1 and TCF7L2 in CRC specimens from patients who progressed on cetuximab and patients with metastatic disease were analyzed by RNAscope and immunohistochemical staining.

**Results:**

The expression of MIR100HG was strongly correlated with EMT markers and acted as a positive regulator of EMT. MIR100HG sustained cetuximab resistance and facilitated invasion and metastasis in CRC cells both in vitro and in vivo. hnRNPA2B1 was identified as a binding partner of MIR100HG. Mechanistically, MIR100HG maintained mRNA stability of TCF7L2, a major transcriptional coactivator of the Wnt/β-catenin signaling, by interacting with hnRNPA2B1. hnRNPA2B1 recognized the N6-methyladenosine (m^6^A) site of TCF7L2 mRNA in the presence of MIR100HG. TCF7L2, in turn, activated MIR100HG transcription, forming a feed forward regulatory loop. The MIR100HG/hnRNPA2B1/TCF7L2 axis was augmented in specimens from CRC patients who either developed local or distant metastasis or had disease progression that was associated with cetuximab resistance.

**Conclusions:**

MIR100HG and hnRNPA2B1 interact to control the transcriptional activity of Wnt signaling in CRC via regulation of TCF7L2 mRNA stability. Our findings identified MIR100HG as a potent EMT inducer in CRC that may contribute to cetuximab resistance and metastasis by activation of a MIR100HG/hnRNPA2B1/TCF7L2 feedback loop.

**Supplementary Information:**

The online version contains supplementary material available at 10.1186/s12943-022-01555-3.

## Introduction

Epithelial-to-mesenchymal transition (EMT) is a dynamic biological process by which epithelial cells lose cell-cell contacts, apical-basal polarity and distinct cytoskeletal architecture to become more motile and invasive [1]. Epithelial cancer cells undergoing EMT can transition to a mesenchymal phenotype characterized by enhanced drug resistance and metastatic ability [2]. Colorectal cancer (CRC) is the third leading cause of new cancer cases and the second leading cause of cancer-related deaths worldwide [3]. Drug resistance and metastasis occur all too often in CRC patients and are major contributors to poor outcomes [4]. In the clinic, anti-epidermal growth factor receptor (EGFR)-based therapies are frequently used to treat CRC patients with metastatic disease if the tumors are wild-type (WT) *KRAS* [5]. However, the effectiveness of anti-EGFR monoclonal antibodies such as cetuximab and panitumumab is often limited due to de novo and acquired drug resistance [6]. Although inherent genetic mechanisms encompassing mutations in *KRAS*/*NRAS*/*BRAF* and amplification of *ERBB2* and *MET* have primarily been identified as conferring resistance to anti-EGFR treatment, about 30% of cases that are unresponsive to anti-EGFR therapies arise from unknown, apparently non-genetic resistance mechanisms [6]. EMT represents a pivotal program that can be hijacked by cancer cells and is associated with the acquisition of tumor invasiveness and therapeutic resistance, but its involvement in cetuximab resistance in CRC is incompletely understood.

We previously identified that two microRNAs (miRNAs), miR-100 and miR-125b, act in concert to activate Wnt signaling via repression of five Wnt/β-catenin negative regulators that contribute to cetuximab resistance in CRC [7]. Both miR-100 and miR-125b are embedded in the third intron of the gene *MIR100HG*, which encodes the long non-coding RNA (lncRNA) MIR100HG. MIR100HG is referred to as a miRNA-host gene lncRNA (lnc-MIRHG), a subclass of newly discovered lncRNAs derived from miRNA host genes due to pre-miRNA processing [8]. It is estimated that about 17.5% of miRNAs are generated by lnc-MIRHGs [9]. Although the functions of the encoded miRNAs are usually well studied, determining if independent roles exist for host lnc-MIRHGs is an open area of investigation. Recent studies have begun to converge on miRNA-independent roles of MIR100HG in cancer. It has been reported that MIR100HG regulates the cell cycle by facilitating an interaction between the RNA-binding protein HuR and its target mRNAs [10]. MIR100HG was also found to promote breast cancer cell proliferation by forming an RNA–DNA triplex with the p27 locus [11]. These findings imply that MIR100HG plays an active role in cancer progression and is not merely a precursor or non-functional byproduct of miRNA processing. However, the biological function and underlying mechanism of MIR100HG in cetuximab resistance in CRC has not been studied.

N6-methyladenosine (m^6^A) is one of the most prevalent internal modifications of eukaryotic mRNAs [12]. The presence of m^6^A on transcripts affects a diverse set of fundamental cellular processes, including pre-mRNA splicing, nuclear transport, mRNA stabilization and translation [13, 14]. The addition of a m^6^A modification is a dynamic multistep process introduced by the m^6^A methyltransferases (termed “writers”), removed by the demethylases (termed “erasers”) and recognized by m^6^A binding proteins (termed “readers”) [15]. Recent studies indicated that m^6^A modifications of mRNAs are increased in cancer cells during EMT, and that deletion of the m^6^A writer, METTL3, can impair EMT, while deletion of the m^6^A eraser, FTO, can promote EMT [16, 17]. lncRNAs are reported to participate in all steps of m^6^A processing, including methylation, demethylation and recognition via binding to diverse m^6^A modifiers [18–20]. Although it is established that lncRNAs are critical regulators of EMT [21], whether their effects on EMT are mediated by modulating m^6^A modification is unknown. Heterogeneous nuclear ribonucleoprotein A2B1 (hnRNPA2B1) is a member of the hnRNP family and is involved in RNA transcription, stabilization, splicing and translation [22]. hnRNPA2B1 has been found to play a direct role in cancer development and progression by inducing EMT in various cancer types [23–25]. hnRNPA2B1 has been proposed as a m^6^A reader involved in primary miRNA processing and the innate immune response to DNA viruses [26, 27]. Recent evidence suggests that hnRNPA2B1 may mediate effects of m^6^A through a “m^6^A switch” mechanism [28]. However, it remains unknown whether hnRNPA2B1 modulates the EMT process via regulation of m^6^A modifications.

Here, we demonstrate that MIR100HG exerts a miRNA-independent role in regulating EMT, cetuximab resistance and metastasis in CRC cells by interacting with hnRNPA2B1. MIR100HG and hnRNPA2B1 cooperatively enhance the mRNA stability of TCF7L2, a downstream effector of Wnt signaling, in a m^6^A-dependent manner, which facilitates expression of Wnt target genes. TCF7L2 activates MIR100HG transcription, creating a feed forward regulatory loop. The MIR100HG/hnRNPA2B1/TCF7L2 axis was augmented in CRC specimens from patients who progressed on cetuximab and those with metastasis. Collectively, our findings reveal an actionable, epigenetic cause of cetuximab resistance and metastasis in advanced CRC involving a MIR100HG/hnRNPA2B1/TCF7L2 feedback loop.

## Materials and methods

### Cell culture

The human CRC cell lines NCI-H508, Caco-2, SW403, SW948, HT29, SK-CO-1, DLD-1, SW480, SW837, SW48, SW620, LoVo, COLO205, T84, LS174T, NCIH716, HCT8, HCT15, SW1116, RKO, COLO320DM, HuTu80, LS123, HCT116, DiFi, GEO, LIM1215, LIM2405 and V9P were used as previously described [7]. Cetuximab-sensitive CC cells and cetuximab-resistant CC-CR cells were generated by culturing the CRC cell line, HCA-7, in a three-dimensional (3D) system with type-1 collagen and continuous cetuximab (3 μg/ml) treatment for approximately 4 months [7]. All cells were cultured in Dulbecco’s modified Eagle’s medium (DMEM, Gibco) supplemented with 10% fetal bovine serum (Gibco), glutamine (Gibco), nonessential amino acids (Gibco), 100 U/ml penicillin and 100 μg/ml streptomycin (HyClone) in 5% CO_2_ at 37 °C. All cell lines were confirmed to be free of mycoplasma contamination. 3D collagen cultures were performed as previously described [7].

### RNAscope assay

MIR100HG and TCF7L2 mRNA expression was detected by in situ hybridization (ISH) using an RNAscope Multiplex Fluorescent Kit V2 combined with immunofluorescence on formalin-fixed paraffin-embedded (FFPE) tissue sections according to the manufacturer’s instructions (Advanced Cell Diagnostics). The MIR100HG (No. 483151) and TCF7L2 probes (No. 420041) were purchased from Advanced Cell Diagnostics. A confocal microscope (Nikon A1R) was used for image analysis. RNAscope staining was categorized into five grades 0, 1, 2, 3 and 4, according to the following criteria: 0, no staining or less than 5% tumor cells in each field examined; 1, 5 ~ 10% tumor cells stained in each field examined; 2, 10 ~ 25% tumor cells stained in each field examined; 3, 25 ~ 50% tumor cells stained in each field examined; 4, 50 ~ 100% tumor cells stained in each field examined. A score ≥ 2 was considered positive. Three fields of a tissue section were selected for histology quantification. Spearman rank correlation was adopted for statistical analyses of the association between target genes of interest.

A detailed description of the Materials and Methods used in this study can be found in Supplementary Material.

## Results

### MIR100HG is closely related to and involved in the regulation of EMT in CRC

**Fig. 1:**
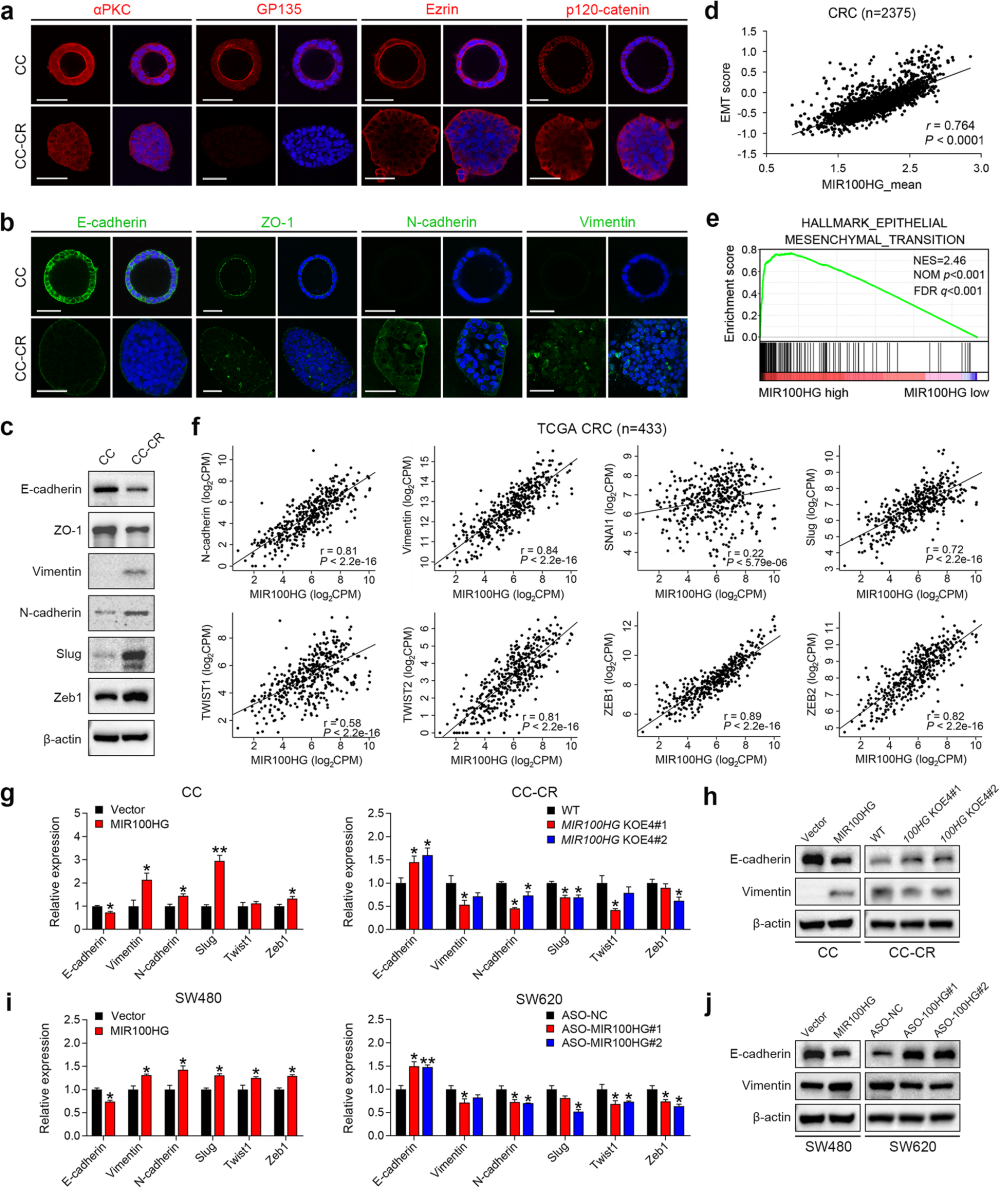
Regulation of EMT in CRC by MIR100HG. **a** Immunofluorescence of αPKC, GP135, Ezrin and p120-catenin in CC and CC-CR cells in 3D culture. **b** Immunofluorescence of E-cadherin, ZO-1, N-cadherin and Vimentin in CC and CC-CR cells in 3D culture. **c** Immunoblots of indicated proteins from 3D-cultured CC and CC-CR cell lysates. β-actin served as a loading control. Representative of three independent experiments. **d** Correlation analysis of MIR100HG expression and EMT score in CRC patients (*n* = 2375). **e** GSEA of an EMT gene signature in CRC samples with high MIR100HG expression versus those with low MIR100HG expression in TCGA database (*n* = 478). The median expression of MIR100HG was used as the cut-off. **f** Correlation analyses of expression of MIR100HG and EMT markers in TCGA database (*n* = 433). **g-j** qPCR and immunoblots of EMT markers in indicated cells, *n* = 3 independent biological replicates. **P < 0.01, *P < 0.05. Data represent mean ± s.d

We previously described the generation of cetuximab-sensitive CC cells and cetuximab-resistant CC-CR cells from human HCA-7 cells, a WT *KRAS*/*NRAS*/*BRAF*, microsatellite unstable CRC cell line [7]. CC cells seeded in 3D culture using type-1 collagen generated hollow, fully polarized structures, as indicated by apical localization of atypical PKC, GP135, Ezrin and lateral localization of p120-catenin. In contrast, the majority of CC-CR cells exhibited solid, disorganized structures with loss of apico-basalateral polarity (Fig. 1a). The loss of cell polarity is considered a hallmark of cancer progression with epithelial cells undergoing a mesenchymal transition as an important step [1]. CC-CR cells showed a loss of E-cadherin-mediated adherens junctions, reduction of the tight junction protein, ZO-1, and increased expression of mesenchymal markers, N-cadherin and vimentin, compared to CC cells (Fig. 1b, c and Supplementary Fig. 1a). Expression of EMT-inducing transcription factors (EMT-TFs), including Snail, Twist and ZEB family members, were also elevated in CC-CR cells (Fig. 1c and Supplementary Fig. 1a). These features suggest EMT occurs in CC-CR cells in 3D culture.

Since MIR100HG is the most overexpressed transcript in CC-CR compared to CC, we thus investigated whether MIR100HG was involved in EMT. We first analyzed the correlation between MIR100HG and a list of EMT gene expression signatures [29] in a large human CRC data set (*n* = 2375) and found that MIR100HG and EMT were highly correlated (Fig. 1d, Pearson *R* = 0.764, *P* < 0.0001). By employing an EMT hallmark gene set from the Molecular Signatures Database (MSigDB) [30], we demonstrated that EMT gene expression patterns were significantly enriched in CRC samples with a high level of MIR100HG by Gene Set Enrichment Analysis (GSEA) (*n* = 478, Fig. 1e). Furthermore, analysis of The Cancer Genome Atlas (TCGA) CRC data repository (*n* = 433) revealed that there were strong positive correlations between MIR100HG and the expression of mesenchymal genes or EMT-TFs (Fig. 1f). In addition, weak but significant negative correlations between MIR100HG and the expression of epithelial genes were also observed (Supplementary Fig. 1b). These results strongly suggest that MIR100HG expression is correlated with a mesenchymal state of CRC.

MIR100HG is encoded by four exons (3129 bp in length) of the *MIR100HG* gene, which spans 394 kb of chromosome. To determine whether MIR100HG regulates the EMT process in CRC cells, we specifically deleted the longest exon, exon 4, of *MIR100HG* (2638 bp) using CRISPR/Cas9 gene-editing (Supplementary Fig. 1c) in CC-CR cells; the resulting line is designated *MIR100HG*^KOE4^. We also overexpressed MIR100HG in CC cells by transduction of a lentiviral vector expressing the full-length MIR100HG transcript (Supplementary Fig. 1d). qPCR confirmed that cells with depleted or overexpressed MIR100HG did not show a significant change in levels of the *MIR100HG* intron-encoded miR-100 or miR-125b (Supplementary Fig. 1d). MIR100HG-overexpressing CC cells showed reduced expression of E-cadherin and increased expression of mesenchymal genes and EMT-TFs; in marked contrast, increased expression of these genes was observed in *MIR100HG*^KOE4^ cells (Fig. 1g and h). Similar results were observed in SW480 (low endogenous MIR100HG expression) and SW620 (moderate endogenous MIR100HG expression) cells after MIR100HG overexpression or knockdown by transfection with antisense oligonucleotides (ASOs), respectively (Fig. 1i, j and Supplementary Fig. 1d, e). Collectively, these results indicate that MIR100HG induces EMT in CRC.

### MIR100HG is important for maintaining cetuximab resistance and promoting metastasis in CRC cells

**Fig. 2:**
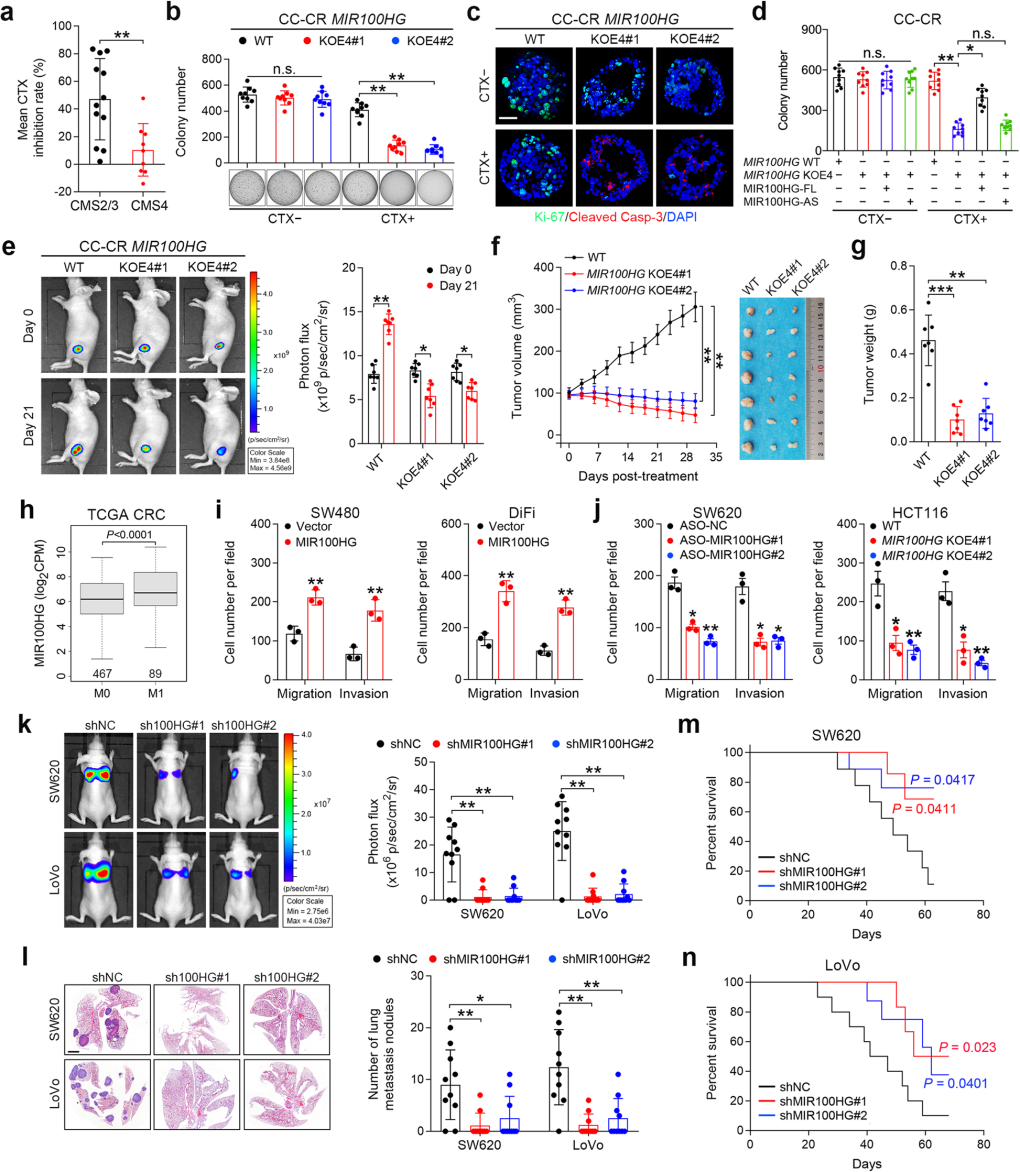
MIR100HG drives cetuximab resistance and metastasis in CRC cells. **a** Mean CTX inhibition rate of CRC cells categorized by the gene expression–based consensus molecular subtyping (CMS). **b** Colony counts for 3D-cultured *MIR100HG*^KOE4^ cells in the presence or absence of CTX (3 μg/ml) after 18 d (*n* = 3 independent experiments performed in triplicate). **c** Representative images of Ki-67 (green) and cleaved Caspase-3 (red) staining in *MIR100HG*^KOE4^ CC-CR cells treated with CTX (10 μg/ml) for 24 h after culturing in 3D culture for 12 d, *n* = 5 independent experiments. Scale bar, 50 μm. **d** Colony counts for 3D-cultured CC-CR cells with indicated treatment (*n* = 3 independent experiments performed in triplicate). **e** Representative bioluminescence images of athymic nude mice (*n* = 7) injected subcutaneously with WT or *MIR100HG*^KOE4^ CC-CR cells that received CTX treatment (1 mg per mouse, intraperitoneal (i.p.) injection every 3 d). **f, g** Growth curve with image (**f**) and weight (**g**) of tumors in athymic nude mice (*n* = 7) injected with indicated cells. **h** Expression of MIR100HG in CRC patients with (M1) or without (M0) metastasis from TCGA database. **i, j** Extent of migration or invasion using Transwell migration and invasion assays of indicated cells when MIR100HG was overexpressed (**i**) and knocked down or knocked out (**j**). *n* = 3 independent biological replicates. **k** Representative bioluminescence images of indicated groups of mice (*n* = 10) at 8 weeks after tail vein injection (left) as well as radiance measurements (right). **l** Representative hematoxylin and eosin (H&E)-stained images of lung tissue sections from different groups (left). The number of lung metastatic foci was calculated (right) (*n* = 10). Scale bars, 200 μm. **m, n** Overall survival for mice in indicated groups. ***P < 0.001, **P < 0.01, *P < 0.05. Data represent mean ± s.d., n.s., not significant

We next investigated whether MIR100HG is involved in drug resistance and metastasis in CRC, phenotypes closely associated with EMT [2, 31]. We first analyzed cetuximab responsiveness in 29 CRC cell lines stratified by gene expression-based consensus molecular subtyping (CMS) [32] (Supplementary Table 9). We found that cells in the CMS2 and CMS3 classifications were responsive to cetuximab treatment, while cells in the CMS4 mesenchymal group exhibited cetuximab resistance (Fig. 2a and Supplementary Fig. 2a), supporting that EMT confers cetuximab resistance in CRC. In 3D culture, *MIR100HG*^KOE4^ CC-CR cells showed comparable colony numbers to the WT cells; however, in the presence of cetuximab, *MIR100HG*^KOE4^ cells showed a significant reduction in colony numbers compared to WT (Fig. 2b). Decreased Ki-67 and increased cleaved caspase-3 staining were observed in *MIR100HG*^KOE4^ CC-CR cells following cetuximab treatment (Fig. 2c and Supplementary Fig. 2b). Restoration of the full-length MIR100HG transcript (MIR100HG-FL), but not the antisense one (MIR100HG-AS), largely abrogated responsiveness to cetuximab in *MIR100HG*^KOE4^ CC-CR cells (Fig. 2d). To determine whether these findings could be recapitulated in vivo, we established subcutaneous xenografts in athymic nude mice with *MIR100HG*^KOE4^ CC-CR cells transduced with a luciferase-expressing lentiviral vector and then treated the mice with cetuximab (Fig. 2e). The in vivo bioluminescence, as well as tumor volumes and weights, in the *MIR100HG*^KOE4^ group after cetuximab treatment were markedly decreased compared to the control group (Fig. 2f and g). Immunohistochemical (IHC) staining showed that *MIR100HG*^KOE4^ tumors treated with cetuximab contained fewer Ki-67-positive cells and more cleaved caspase-3-positive cells (Supplementary Fig. 2c). These results indicate that MIR100HG is important for maintenance of cetuximab resistance in CRC cells and that reducing the levels of MIR100HG can restore drug sensitivity.

We next determined whether MIR100HG contributes to CRC metastasis. Analysis of the TCGA data repository revealed that MIR100HG expression is significantly higher in CRC patients with metastasis than in patients without metastasis (Fig. 2h). Upregulation of MIR100HG in SW480 cells, as well as in DiFi cells (low endogenous MIR100HG expression; Supplementary Fig. 1e), significantly increased migration and invasion (Fig. 2i and Supplementary Fig. 2d). In contrast, knockdown of MIR100HG in SW620 cells, as well as in HCT116 cells (high endogenous MIR100HG expression; Supplementary Fig. 1e), hindered their migration and invasion abilities (Fig. 2j and Supplementary Fig. 2e). Furthermore, we employed in vivo metastasis assays by injecting MIR100HG-silenced SW620 or LoVo (moderate endogenous MIR100HG expression; Supplementary Fig. 1e) cells into the tail vein of nude mice. Compared to the control group, mice injected with MIR100HG-silenced cells showed a decrease in metastasis, as indicated by the overall bioluminescence signal and number of lung metastasis, and improved survival (Fig. 2k-n and Supplementary Fig. 2f). These results indicate that MIR100HG promotes tumor invasion and enhances metastatic capability in CRC cells.

### hnRNPA2B1 is a direct and functional binding partner of MIR100HG

**Fig. 3:**
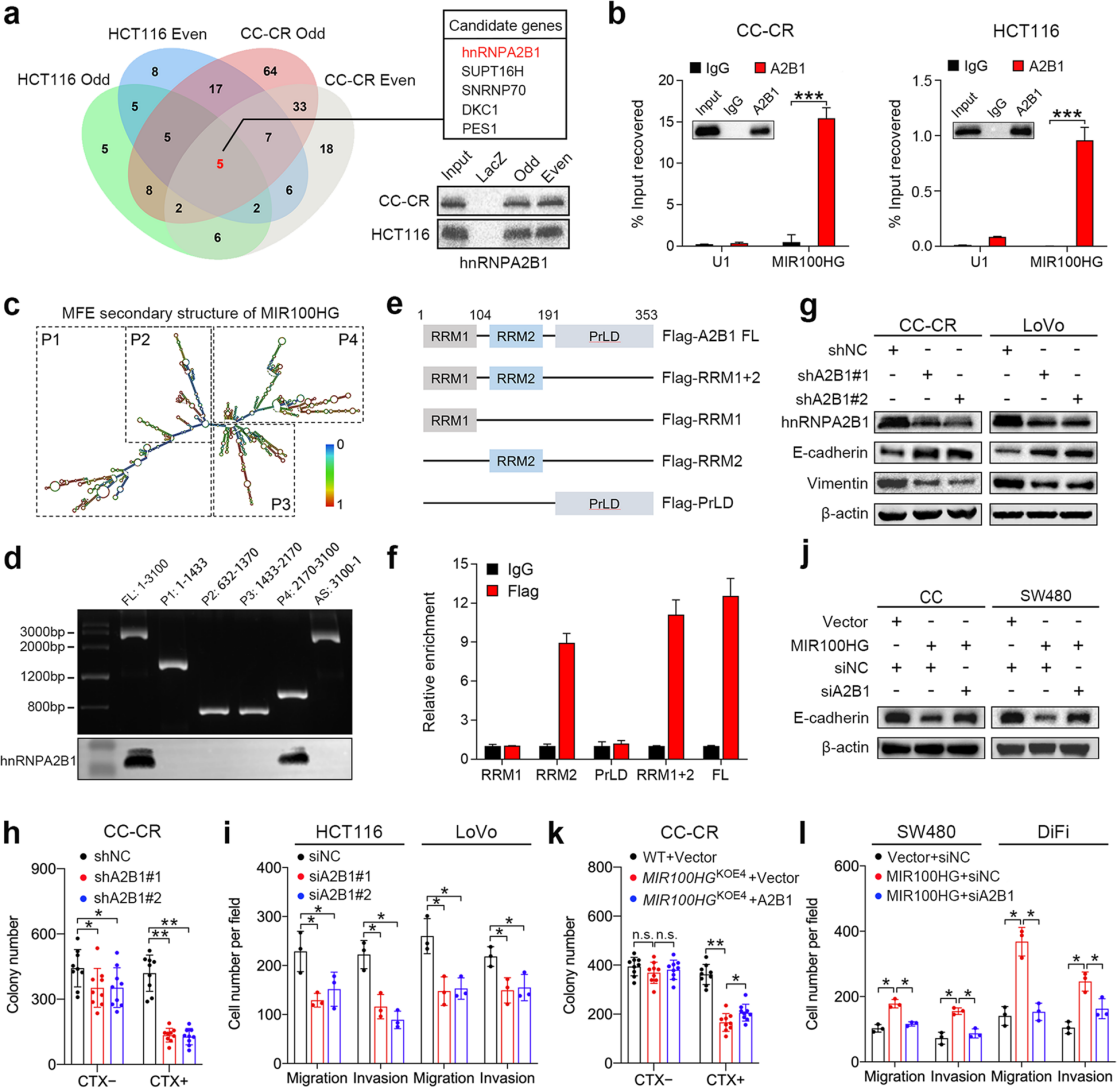
hnRNPA2B1 is a direct and functional binding partner of MIR100HG. **a** Venn diagram proteins identified by mass spectrometry and retrieved by different groups of MIR100HG probes in ChIRP assays. “Even” and “odd” indicate the two groups of biotin-labeled oilgo probe sets designed for MIR100HG. Immunoblots of hnRNPA2B1 enriched by MIR100HG probes in CC-CR and HCT116 cells (bottom right). **b** RIP assays detecting MIR100HG retrieved by a hnRNPA2B1 specific antibody or by normal IgG. U1 RNA served as a negative control. Inset depicts immunoblots showing pull-down efficiency. *n* = 3 independent biological replicates. **c** Graphic illustration of the predicted secondary structure of MIR100HG. **d** Top: agarose gel electrophoresis analysis of full-length (FL), truncated or antisense (AS) MIR100HG transcripts; Bottom: immunoblots for hnRNPA2B1 pulled-down by the indicated transcripts of MIR100HG. Representative of three independent experiments. **e** Diagrams of FL and domain-truncated fragments of hnRNPA2B1. RRM, RNA recognition motif; PrLD, prion-like domain. **f** Detection of MIR100HG by RIP assay as retrieved by Flag-tagged FL or domain-truncated hnNRNPA2B1 using a Flag antibody. An IgG antibody served as a negative control. *n* = 3 independent biological replicates. **g** Immunoblots of indicated EMT markers in CC-CR and LoVo cells after hnRNPA2B1 knockdown. Representative of three independent experiments. **h** Colony counts of 3D-cultured CC-CR cells after hnRNPA2B1 knockdown in the presence or absence of CTX (3 μg/ml), *n* = 3 independent experiments performed in triplicate. **i** Extent of migration and invasion using Transwell migration and invasion assays for indicated cells after hnRNPA2B1 knockdown. *n* = 3 independent biological replicates. **j** Immunoblots for E-cadherin in CC and SW480 cells with indicated treatment. Representative of three independent experiments. **k** Colony counts of 3D-cultured *MIR100HG*^KOE4^ CC-CR cells transduced with hnRNPA2B1-expressing lentiviral particles in the presence or absence of CTX (3 μg/ml), *n* = 3 independent experiments performed in triplicate. **l** Extent of migration and invasion using Transwell migration and invasion assays for SW480 and DiFi cells with the indicated treatment. *n* = 3 independent biological replicates. ***P < 0.001, **P < 0.01, *P < 0.05. Data represent mean ± s.d., n.s., not significant

One of the ways that lncRNAs can exert their effects is by directly interacting with RNA binding proteins [33]. Since MIR100HG is mainly located in the nucleus, we performed chromatin isolation by RNA purification (ChIRP) coupled to mass spectrometry-based profiling to identify nuclear-localized interacting partners of MIR100HG in CRC cells. In total, 173 and 76 ChIRP-purified proteins were isolated from CC-CR and HCT116 cells, respectively, using two groups of biotin-labeled oligo probe sets targeting MIR100HG (Fig. 3a; Supplementary Table 8 and 10). Among them, five candidates, hnRNPA2B1, SUPT16H, SNRNP70, DKC1 and PES1, were enriched in all groups (Fig. 3a). hnRNPA2B1 was chosen for further investigation as it has been reported to be an EMT regulator in various cancer types [23–25]. The enrichment of hnRNPA2B1 by MIR100HG probes was confirmed in CC-CR and HCT116 cells (Fig. 3a) and further validated by RNA-binding protein immunoprecipitation (RIP) assays which showed that MIR100HG was significantly enriched in pull-downs using antibodies against hnRNPA2B1 compared to control IgG (Fig. 3b). To determine the specific region of MIR100HG that binds to hnRNPA2B1, a series of MIR100HG fragments were generated based on its secondary structure predicted by the RNAfold web server (Fig. 3c) and then these constructs were used in biotin-labeled RNA pull-down assays. The results showed that hnRNPA2B1 mainly binds to a MIR100HG fragment that is transcribed from nucleotides 2170 to 3100 (Fig. 3d). In addition, RIP assays using antibodies against full-length or truncated hnRNPA2B1 with a Flag-tag were carried out to elucidate the specific domain of hnRNPA2B1 that mediates the interaction with MIR100HG (Fig. 3e). The results revealed that the RNA recognition motif 2 (RRM2) domain of hnRNPA2B1 was primarily responsible for the interaction with MIR100HG (Fig. 3f). These results indicate that hnRNPA2B1 is a bona fide interacting partner of MIR100HG in CRC cells.

We then investigated the role of hnRNPA2B1 in CRC progression. Silencing of hnRNPA2B1 increased E-cadherin and reduced vimentin expression in CC-CR and LoVo cells (relatively high endogenous MIR100HG and hnRNPA2B1 expression; Supplementary Fig. 1e and 3a), consistent with the effect of MIR100HG on EMT (Figs. 1h, h and 3g). In 3D culture, knockdown of hnRNPA2B1 in CC-CR cells reduced total colony numbers with the reduction being more pronounced with cetuximab treatment, indicating that hnRNPA2B1 can contribute to cetuximab resistance (Fig. 3h). Meanwhile, knockdown of hnRNPA2B1 markedly hindered cell migration and invasion (Fig. 3i and Supplementary Fig. 3b). We next sought to elucidate the role of hnRNPA2B1 in MIR100HG-mediated cetuximab resistance and metastasis. Silencing of hnRNPA2B1 reversed E-cadherin reduction, which was mediated by MIR100HG overexpression in CC and SW480 cells (Fig. 3j). In the presence of cetuximab, *MIR100HG*^KOE4^ CC-CR cells exhibited reduced colony numbers, which was partially offset by hnRNPA2B1 overexpression (Fig. 3k). Knockdown of hnRNPA2B1 also abolished the enhanced migration and invasion ability of MIR100HG-overexpressing cells (Fig. 3l and Supplementary Fig. 3c). Collectively, these results suggest that MIR100HG promotes CRC progression via its interaction with hnRNPA2B1.

### MIR100HG and hnRNPA2B1 regulate TCF7L2 mRNA stability and activate Wnt signaling

**Fig. 4:**
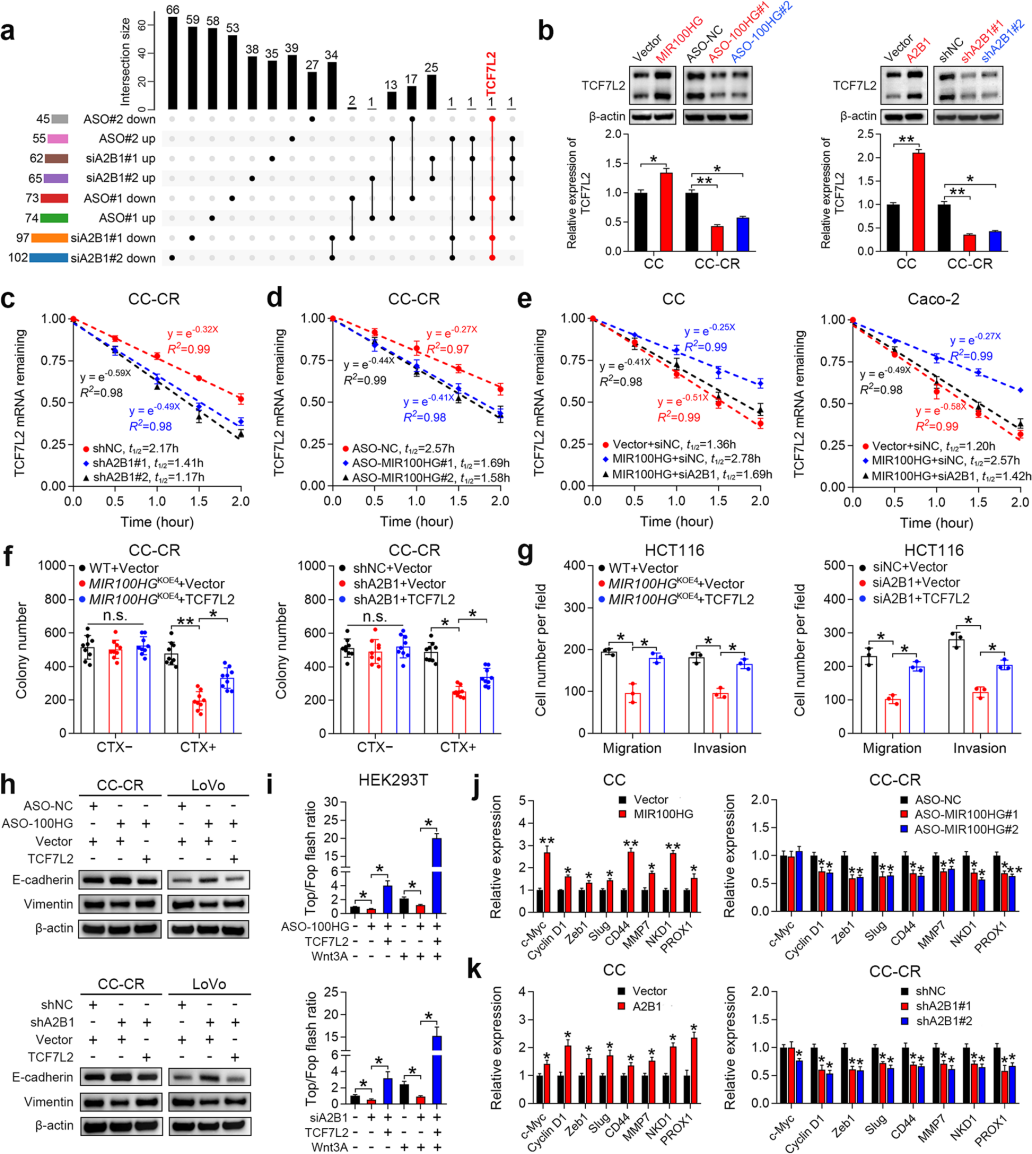
MIR100HG and hnRNPA2B1 collaboratively regulate TCF7L2 mRNA stability and activate Wnt signaling. **a** Upset plot showing the number of significantly up- or down-regulated genes in multiple comparisons of the RNA-sequencing data of HCT116 cells after MIR100HG or hnRNPAB1 knockdown. **b** Immunoblots and qPCR analyses of TCF7L2 expression in CC and CC-CR cells after manipulation of MIR100HG (left) or hnRNPA2B1 (right) expression. Representative of three independent experiments. **c, d** Assessment of TCF7L2 mRNA half-life (t_1/2_) in hnRNPA2B1- (**c**) or MIR100HG- (**d**) silenced CC-CR cells. *n* = 3 independent biological replicates. **e** Assessment of TCF7L2 mRNA half-life in MIR100HG-overexprssing CC and Caco-2 cells after hnRNPA2B1 knockdown. *n* = 3 independent biological replicates. **f** Colony counts for 3D-cultured *MIR100HG*^KOE4^ (left) or hnRNPA2B1-silenced (right) CC-CR cells after TCF7L2 overexpression in the presence or absence of CTX (3 μg/ml), *n* = 3 independent experiments performed in triplicate. **g** Extent of migration and invasion using Transwell migration and invasion assays for *MIR100HG*^KOE4^ (left) or hnRNPA2B1-silenced (right) HCT116 cells after TCF7L2 overexpression. *n* = 3 independent biological replicates. **h** Immunoblots of E-cadherin and vimentin in CC-CR and LoVo cells after the indicated treatment. Representative of three independent experiments. **i** Top/Fop flash luciferase reporter activity in HEK293T cells after indicated treatment. *n* = 3 independent biological replicates. **j** qPCR analyses of Wnt target genes in CC and CC-CR cells after manipulation of MIR100HG (**j**) or hnRNPA2B1 (**k**) expression. *n* = 3 independent biological replicates. **P < 0.01, *P < 0.05. Data represent mean ± s.d., n.s., not significant

To explore how MIR100HG contributes to CRC progression through binding to hnRNPA2B1, we first examined whether MIR100HG affects hnRNPA2B1 expression. Neither overexpression nor knockdown of MIR100HG influenced mRNA or protein levels of hnRNPA2B1 in CRC cells (Supplementary Fig. 4a-c). Since hnRNPA2B1 shuttles between the nucleus and the cytoplasm [34], we analyzed the subcellular distribution of hnRNPA2B1 in MIR100HG-overexpressing and silenced cells and found that MIR100HG had no influence on cellular distribution of hnRNPA2B1 (Supplementary Fig. 4d and e). Given that hnRNPs are associated with precursor mRNAs (pre-mRNAs) and influence pre-mRNA processing and metabolism [22], we speculated that certain mRNAs might be affected by the interaction between MIR100HG and hnRNPA2B1. To profile mRNA candidates regulated by both MIR100HG and hnRNPA2B1, we silenced either MIR100HG or hnRNPA2B1 and performed RNA sequencing. Notably, TCF7L2 (also known as TCF4) was identified as the only significantly downregulated gene (fold-change > 2, *p* < 0.05) consistent across all MIR100HG- and hnRNPA2B1-silenced groups (Fig. 4a). Since our previous study revealed that a positive correlation exists between MIR100HG and Wnt signaling [7], we decided to focus efforts on TCF7L2 since it is a critical transcriptional coactivator of Wnt signaling, which is tightly linked to drug resistance, metastasis and EMT [35]. As shown in Fig. 4b, knockdown of MIR100HG or hnRNPA2B1 reduced TCF7L2 mRNA and protein expression in CC-CR and HCT116 cells (high endogenous MIR100HG and hnRNPA2B1 expression; Supplementary Fig. 1e, 3a, 4f and 4g), while overexpression of MIR100HG or hnRNPA2B1 increased TCF7L2 mRNA and protein levels in CC and SW480 cells (relatively low endogenous MIR100HG and moderate hnRNPA2B1 expression; Supplementary Fig. 1a, 3a, 4f and 4g). However, manipulation of MIR100HG or hnRNPA2B1 did not affect the transcription of TCF7L2, as evidenced by the unchanged levels of pre-mRNA of TCF7L2 (Supplementary Fig. 4h), suggesting that TCF7L2 is regulated post-transcriptionally by MIR100HG and hnRNPA2B1.

hnRNPs are known regulators of RNA stability [22]. To test whether hnRNPA2B1 could affect the stability of TCF7L2 mRNA, we treated cells with Actinomycin D to halt new RNA synthesis and examined TCF7L2 mRNA levels. Accelerated mRNA decay of TCF7L2 upon hnRNPA2B1 knockdown was confirmed in CC-CR and HCT116 cells (Fig. 4c and Supplementary Fig. 4i), indicating that hnRNPA2B1 acts to stabilize TCF7L2 mRNA. Knockdown of MIR100HG also enhanced TCF7L2 mRNA degradation (Fig. 4d and Supplementary Fig. 4j). Overexpression of MIR100HG extended the half-life of TCF7L2 mRNA in CC and Caco-2 cells, but silencing of hnRNPA2B1 abrogated the stabilizing effect of MIR100HG (Fig. 4e). These results indicate that hnRNPA2B1 and MIR100HG cooperatively upregulate TCF7L2 expression by enhancing mRNA stability. We next determined whether TCF7L2 is involved in MIR100HG- or hnRNPA2B1-mediated CRC progression. Overexpression of TCF7L2 in MIR100HG- or hnRNPA2B1-depleted CRC cells rescued cetuximab resistance, cell migration and invasion (Fig. 4f, g and Supplementary Fig. 4k). Restoration of TCF7L2 induced EMT, which was inhibited by MIR100HG or hnRNPA2B1 downregulation, as demonstrated by reduced E-cadherin and increased vimentin expression (Fig. 4h). These results indicate that MIR100HG and hnRNPA2B1 function mainly by TCF7L2 modulation.

We next examined whether the functions of MIR100HG/hnRNPA2B1/TCF7L2 axis are mediated by activating Wnt signaling. A luciferase reporter plasmid harboring three optimal TCF/LEF1-binding sites (TOP-flash) or three mutated TCF/LEF1-binding sites (FOP-flash) was transfected into HEK293T cells. TOP/FOP-flash activity was significantly repressed after MIR100HG or hnRNPA2B1 knockdown, whereas this inhibition was rescued by TCF7L2 overexpression, especially after administration of the canonical Wnt ligand Wnt3A (Fig. 4i). In addition, a subset of Wnt target genes, which are associated with cell growth (c-Myc and Cyclin D1) and EMT (ZEB1 and Slug), was significantly upregulated in MIR100HG- and hnRNPA2B1-overexpressing CRC cells (Fig. 4j, k and Supplementary Fig. 4l, m). In contrast, knockdown of MIR100HG and hnRNPA2B1 led to a reduction of these Wnt target genes in CC-CR and HCT116 cells (Fig. 4j, k and Supplementary Fig. 4l, m). Collectively, these results suggest that MIR100HG enhances the stability of TCF7L2 mRNA via interaction with hnRNPA2B1 and subsequently activates the Wnt signaling pathway.

### Stabilization of TCF7L2 mRNA by hnRNPA2B1 and MIR100HG is m^6^A-dependent

**Fig. 5:**
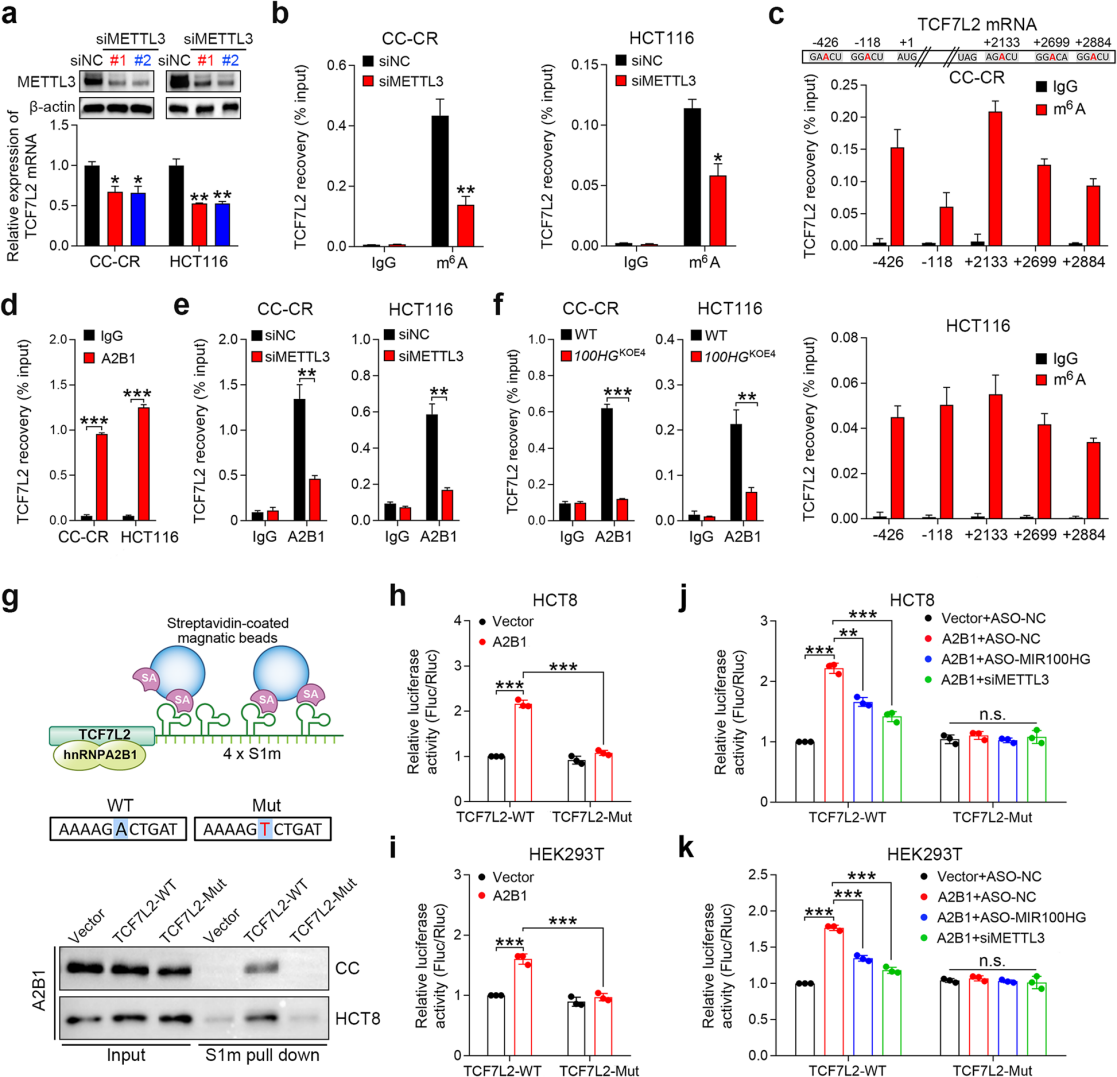
Stabilization of TCF7L2 mRNA by hnRNPA2B1 and MIR100HG is m^6^A dependent. **a** Top, immunoblots of METTL3 showing the silencing efficiency of siRNAs against METTL3. Bottom, qPCR analysis of TCF7L2 expression in CC-CR and HCT116 cells after METTL3 knockdown. *n* = 3 independent biological replicates. **b** Analysis of MeRIP assays detecting TCF7L2 mRNA retrieved by a m^6^A antibody in METTL3-silenced CC-CR and HCT116 cells. *n* = 3 independent biological replicates. **c** Top, graphic illustration of the five high-confidence m^6^A sites predicted by SRAMP in TCF7L2 mRNA. Nucleotide positions are numbered with respect to the transcriptional start site of *TCF7L2*. Bottom, analysis of MeRIP assays detecting TCF7L2 mRNA retrieved by a m^6^A antibody around the five high-confidence m^6^A sites in CC-CR and HCT116 cells. *n* = 3 independent biological replicates. **d** Assessment of RIP assays detecting TCF7L2 mRNA retrieved by a hnRNPA2B1 antibody or by IgG in CC-CR and HCT116 cells. *n* = 2 independent biological replicates. **e, f** Assessment of RIP assays detecting TCF7L2 mRNA retrieved by a hnRNPA2B1 antibody or by IgG in CC-CR and HCT116 cells after METTL3 knockdown (**e**) or *MIR100HG* exon 4 knockout (**f**). *n* = 2 independent biological replicates. **g** Top, schematic representation of the S1m-mediated in vivo pull-down assay. Bottom, immunoblot for hnRNPA2B1 after in vivo expressed WT or mutant S1m tagged TCF7L2 mRNA was pulled down by streptavidin beads. Representative of three independent experiments. **h, i** Luciferase activity of WT (TCF7L2-WT) or mutated (TCF7L2-Mut) TCF7L2 reporters in HCT8 (**h**) and HEK293T (**i**) cells with ectopically expressed hnRNPA2B1. *n* = 2 independent biological replicates. **j, k** Luciferase activity of TCF7L2-WT or TCF7L2-Mut reporters in HCT8 (**j**) and HEK293T (**k**) cells with ectopically expressed hnRNPA2B1, MIR100HG ASO or METTL3 siRNA. *n* = 2 independent biological replicates. ***P < 0.001, **P < 0.01, *P < 0.05. Data represent mean ± s.d., n.s., not significant

In terms of base modifications of mRNA, m^6^A is one of the most abundant alterations, and it can have profound effects on mRNA stability [12]. Since hnRNPA2B1 is a mediator of m^6^A-dependent nuclear RNA processing events [26, 28], we speculated that hnRNPA2B1 might regulate TCF7L2 mRNA stability in a m^6^A-dependent manner. We found that silencing of the m^6^A methyltransferase METTL3 in CC-CR and HCT116 cells decreased TCF7L2 mRNA levels (Fig. 5a). Methylated RNA immunoprecipitation (MeRIP) assays further confirmed that silencing of METTL3 reduced the level of m^6^A modification for TCF7L2 mRNA (Fig. 5b). Five predicted m^6^A sites in the TCF7L2 mRNA were identified using the SRAMP online tool [36] and then validated by MeRIP assays (Fig. 5c). The greatest enrichment of TCF7L2 mRNA after pull-down with antibodies against m^6^A was for the + 2133 site in the 3’UTR near the stop codon (Fig. 5c). These results indicate that TCF7L2 mRNA is m^6^A modified and that this modification stabilizes TCF7L2 mRNA. Next, we determined whether the interaction between hnRNPA2B1 and TCF7L2 depends on the m^6^A modification. RIP assays demonstrated that TCF7L2 mRNA was enriched by the hnRNPA2B1 antibody in CC-CR and HCT116 cells (Fig. 5d), indicating an interaction between hnRNPA2B1 and TCF7L2 mRNA. Knockdown of METTL3 reduced this enrichment (Fig. 5e), suggesting that the m^6^A modification on TCF7L2 mRNA was necessary for its interaction with hnRNPA2B1. Notably, TCF7L2 mRNA precipitated by hnRNPA2B1 was markedly decreased in *MIR100HG*^KOE4^ CC-CR and HCT116 cells (Fig. 5f), implying that the interaction between hnRNPA2B1 and TCF7L2 is also dependent on MIR100HG. Furthermore, we performed an in vivo RNA precipitation assay to validate whether the interaction between hnRNPA2B1 and TCF7L2 is m^6^A-dependent. In this case, TCF7L2 fragments harboring WT or a mutant + 2133 m^6^A modification site were tagged with S1m (Fig. 5g), a modified streptavidin-binding aptamer that acts similar to biotin but with a higher affinity [37]. Utilization of a system where CC and HCT8 cells were transfected with the S1m-tagged TCF7L2 constructs allowed for m^6^A modification to occur in vivo. The streptavidin aptamer-based capture showed that mutation of the m^6^A site for TCF7L2 abrogated association with hnRNPA2B1 (Fig. 5g). This suggests that m^6^A modification of TCF7L2 mRNA is critical for its association with hnRNPA2B1. In addition, we constructed a firefly luciferase reporter carrying the WT or mutant TCF7L2 sequence (Supplementary Fig. 5a). Ectopic hnRNPA2B1 induced a significant increase in luciferase activity of the WT reporter, but this increase was impaired by mutation of the m^6^A site in HCT8 and HEK293T cells (Fig. 5h and i). Consistently, the hnRNPA2B1-mediated increase of luciferase activity was blocked by METTL3 or MIR100HG knockdown (Fig. 5j and k). Collectively, these data suggest that the m^6^A modification of TCF7L2 mRNA is required for its binding to hnRNPA2B1 with MIR100HG serving as an indispensable partner for the interaction.

### TCF7L2 activates MIR100HG transcription and forms a reciprocal positive feedback loop

**Fig. 6:**
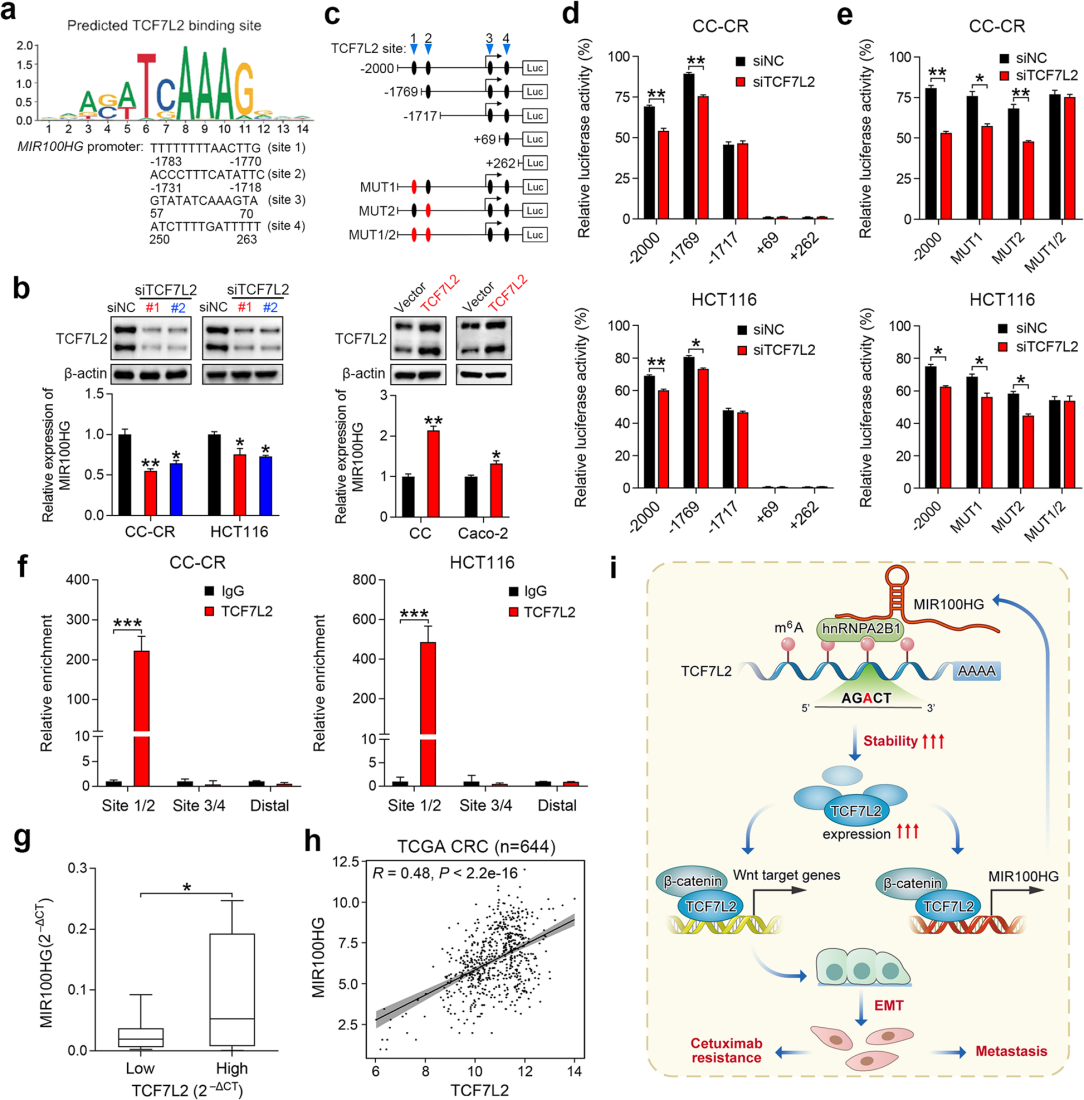
TCF7L2 and MIR100HG forms a reciprocal positive feedback loop in CRC cells. **a** Schematic diagram showing the binding motifs of TCF7L2 in the *MIR100HG* promoter. **b** Top, Immunoblots of TCF7L2 showing the silencing efficiency of siRNAs against TCF7L2. Bottom, qPCR analysis of MIR100HG expression in CC-CR and HCT116 cells after TCF7L2 knockdown. *n* = 3 independent biological replicates. **c** Schematic representation of consecutive deletion or mutation constructs spanning the − 2000 to + 500 region of the *MIR100HG* promoter. Putative TCF7L2-binding sites in the MIR100HG promoter are indicated in black. **d, e** Luciferase activity of serially truncated (**d**) or mutated (**e**) MIR100HG luciferase reporter constructs transfected into CC-CR and HCT116 cells after TCF7L2 knockdown, *n* = 3 independent biological replicates. **f** qPCR assessing the abundance of DNA within the *MIR100HG* promoter region with a primer pair spanning the TCF7L2-binding sites after ChIP assays with a TCF7L2 antibody or control IgG in CC-CR and HCT116 cells. *n* = 3 independent biological replicates. **g** Box plot showing MIR100HG expression in 16 CRC cell lines with low and high TCF7L2 expression. The mean expression of TCF7L2 was used as a cut-off value. **h** Correlation analysis of TCF7L2 and MIR100HG expression in CRC patients from TCGA dataset (*n* = 644). **(i)** Proposed working model in this study. ***P < 0.001, **P < 0.01, *P < 0.05. Data represent mean ± s.d., n.s., not significant

To further explore the mechanism by which MIR100HG is upregulated in CC-CR cells, we analyzed predicted TF binding motifs in the 2-kb promoter region of *MIR100HG* by using the JASPAR database [38]. Of note, four TCF7L2 binding sites were identified in the *MIR100HG* promoter (Fig. 6a), suggesting that MIR100HG may be a TCF7L2-responsive lncRNA. Consistent with this, silencing of TCF7L2 in CC-CR and HCT116 cells (high endogenous TCF7L2 expression; Supplementary Fig. 5b) resulted in reduced MIR100HG expression (Fig. 6b), suggesting that TCF7L2 may be a transcriptional regulator of MIR100HG. In line with this, overexpression of TCF7L2 enhanced MIR100HG expression in CC and Caco-2 cells (low endogenous TCF7L2 expression; Supplementary Fig. 5b) (Fig. 6b). To determine whether TCF7L2 activates MIR100HG transcription, luciferase reporter constructs containing the TCF7L2 binding sites in the *MIR100HG* promoter were transduced into CC-CR and HCT116 cells (Fig. 6c). Serial deletion analysis and site-directed mutagenesis of the *MIR100HG* promoter showed that TCF7L2-binding sites 1 and 2 are the predominant sites for TCF7L2-mediated transcriptional activation (Fig. 6d and e). Chromatin immunoprecipitation (ChIP) assays confirmed that TCF7L2 protein bound directly to the *MIR100HG* promoter at TCF7L2-binding sites 1 and 2 (Fig. 6f). In addition, a positive correlation was observed between TCF7L2 and MIR100HG expression in a panel of CRC cell lines, as well as in the TCGA data repository (Fig. 6g and h). Collectively, these results suggest that a mutual regulatory mechanism exists between MIR100HG and TCF7L2, wherein MIR100HG and hnRNPA2B1 stabilize TCF7L2 mRNA, and, in turn, TCF7L2 elevates MIR100HG abundance via transcriptional activation (Fig. 6i).

### Validation of MIR100HG, hnRNPA2B1 and TCF7L2 expression in CRC specimens

**Fig. 7:**
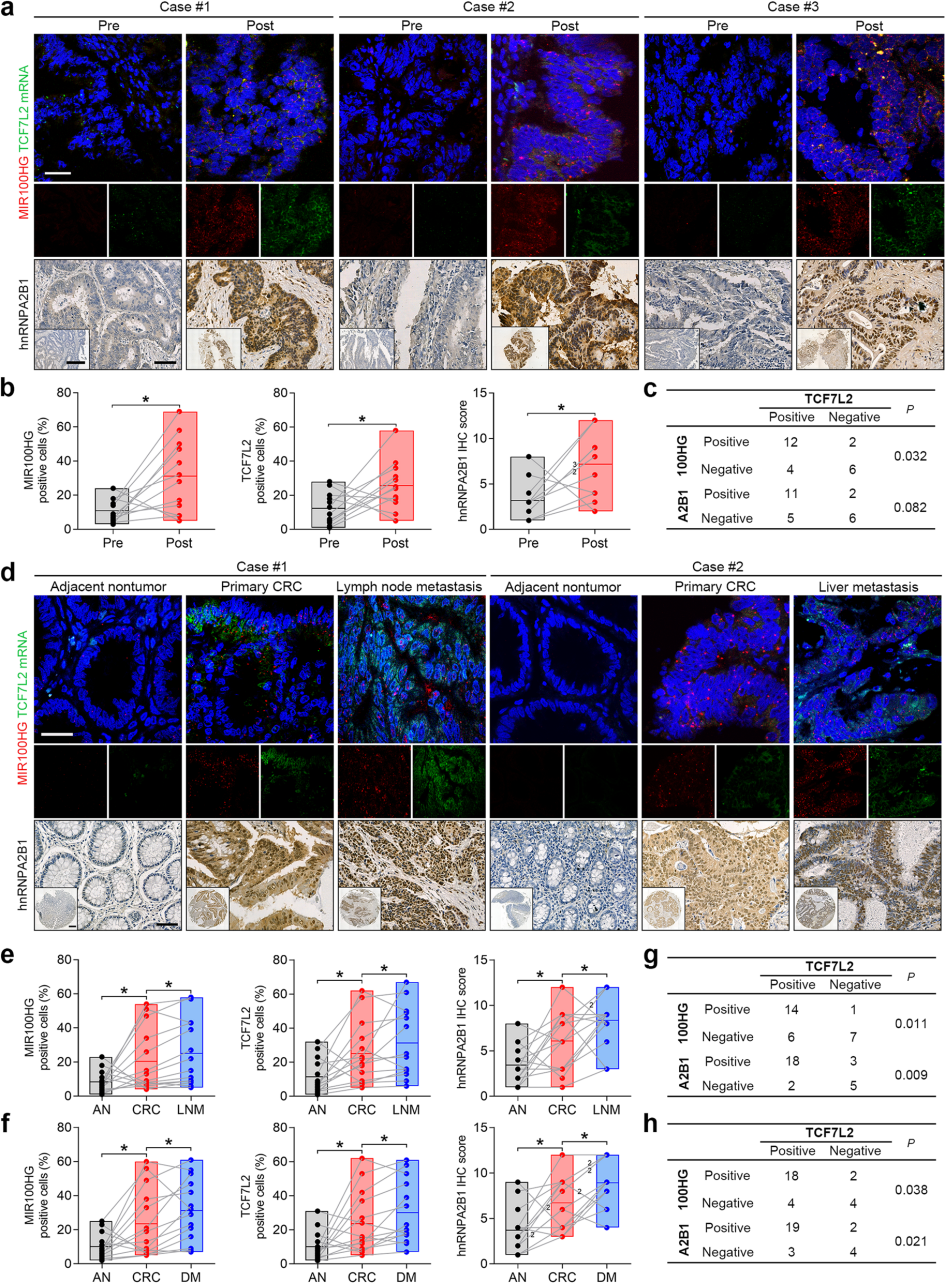
Coordinated expression of MIR100HG/hnRNPA2B1/TCF7L2 in CRC specimens. **a** Representative RNAscope staining images of MIR100HG and TCF7L2 and corresponding IHC staining images of hnRNPA2B1 in paired human CRC specimens obtained pre- and post-cetuximab resistance. Scale bars, 50 μm (main) and 500 μm (inset). **b** Frequency of MIR100HG- (left) and TCF7L2- (middle) positive cells in twelve pairs of matched CRC specimens obtained pre- and post-cetuximab resistance. IHC scores of hnRNPA2B1 in these specimens are shown (right). **c** Correlation analysis of TCF7L2 and MIR100HG or hnRNPA2B1 expression in CRC specimens obtained pre- and post-cetuximab resistance. **d** Representative RNAscope staining images of MIR100HG and TCF7L2 and corresponding IHC staining images of hnRNPA2B1 in paired specimens of primary CRC tissues, adjacent nontumor tissues and their matched lymph node metastases (LNM) or distant metastases (DM). Scale bars, 50 μm (main) and 500 μm (inset). **e, f** Frequency of MIR100HG- (left) and TCF7L2- (middle) positive cells in 14 paired specimens of primary CRC tissues, adjacent nontumor tissues and their matched lymph node (**e**) or distant (**f**) metastases. IHC scores of hnRNPA2B1 in these specimens are shown (right). **g, h** Correlation analysis of TCF7L2 and MIR100HG or hnRNPA2B1 expression in 28 primary (**g**) and metastatic (**h**) CRC tissues. **P < 0.01, *P < 0.05

To examine whether the MIR100HG/hnRNPA2B1/TCF7L2 axis plays a role in cetuximab resistance of CRC patients, we obtained paired tumor specimens from 12 individuals before the start of cetuximab treatment and at the time of tumor progression (Supplementary Table 1). Acquired mutations and amplifications in post-treatment samples were documented if detected (Supplementary Table 11). Using chromogenic RNAscope in situ hybridization, we found that MIR100HG and TCF7L2 were significantly overexpressed in tumors that progressed on cetuximab treatment compared to pre-treatment counterparts (Fig. 7a and b). Increased hnRNPA2B1 immunoreactivity was also observed in serial sections of tumors that progressed on cetuximab (Fig. 7a and b). Positive correlations between MIR100HG or hnRNPA2B1 with TCF7L2 expression were observed (Fig. 7c). These results demonstrate that upregulation of MIR100HG, hnRNPA2B1 and TCF7L2 occurs in the setting of cetuximab resistance in CRC patients.

We next analyzed MIR100HG, hnRNPA2B1 and TCF7L2 expression in 14 paired specimens of primary CRC tissues, adjacent nontumor tissues and their matched lymph node or distant metastasis samples (Supplementary Table 2 and 3). Compared to primary lesions, lymph node, as well as distant metastasis, exhibited significantly higher expression of MIR100HG, hnRNPA2B1 and TCF7L2 (Fig. 7d-f). Positive associations were also identified between MIR100HG or hnRNPA2B1 with TCF7L2 expression in the primary CRC tissues and metastatic lesions (Fig. 7g and h). These results indicate that expression of MIR100HG, hnRNPA2B1 and TCF7L2 is increased in metastatic lesions compared to the primary tumor of these CRC patients.

## Discussion

*MIR100HG* harbors *miR-100*, *let-7a-2* and *miR-125b-1* within its third intron, and hence is referred to as a miRNA host gene. The expression and function of the MIR100HG-embedded miRNAs are well documented [8], but a role for the lncRNA from the *MIR100HG* locus in this context has not been studied. In the present study, we uncovered a novel role for MIR100HG in promoting EMT-mediated cetuximab resistance and metastasis in CRC. MIR100HG and its binding partner, hnRNPA2B1, co-regulate the stabilization of TCF7L2 mRNA, thus enhancing Wnt signaling in CRC cells. We found that the m^6^A modification of the TCF7L2 transcript is a prerequisite for binding to hnRNPA2B1 and that MIR100HG expression is transcriptionally regulated by TCF7L2, thus forming a positive feedback loop. This regulatory circuitry is clinically actionable as verified by the analysis of MIR100HG/hnRNPA2B1/TCF7L2 expression patterns in CRC specimens from patients whose tumors progressed on cetuximab and patients with lymph node or distant metastasis. These data support the potential therapeutic and predictive value of the proposed model.

As an important member of the A/B subfamily of heterogeneous nuclear ribonucleoproteins (hnRNPs), hnRNPA2B1 aids in the transcription, splicing, stability, and translation of a variety of RNA molecules as well as controlling the expression of numerous genes by recognizing and binding specific RNA substrates and DNA motifs [22]. Recent studies demonstrated that hnRNPA2B1 is a common binding partner of lncRNAs and plays a critical role in EMT, drug resistance and metastasis in multiple cancers. In pancreatic cancer, hnRNPA2B1 interacts with linc01232 to accelerate metastasis through A-Raf-induced MAPK/ERK signaling pathway activation [39]. hnRNPA2B1 also reduces the sensitivity of breast cancer cells to chemotherapy drugs by regulating the alternative splicing of Bcl-x pre-mRNA [40]. Our study revealed that hnRNPA2B1 was a direct and functional binding partner of MIR100HG, which adds to the literature describing interactions between hnRNPA2B1 and lncRNAs in the setting of drug resistance and metastasis. TCF7L2 mRNA is stabilized by interaction with hnRNPA2B1 through the assistance of MIR100HG and m^6^A modification.

It should be noted that the mechanism behind hnRNPA2B1 affecting the stability of TCF7L2 mRNA is not fully elucidated. Regulation of mRNA stability depends in part on specific *cis*-acting sequences and trans-acting factors [41]. It has been reported that hnRNPA2B1 regulates mRNA stability by binding UAASUUAU sequences or (U)16 elements in the 3′-UTR of mRNAs [42, 43]. hnRNPA2B1 can also influence mRNA half-life by regulating polyadenylation [44]. Our in vivo pull-down assay revealed that the recognition site between hnRNPA2B1 and TCF7L2 is in the 3′-UTR of TCF7L2 mRNA near the stop codon, providing evidence that the 3′-UTR might be important for this mRNA’s half-life [45]. Understanding the mechanisms underpinning hnRNPA2B1 effects on the stability of TCF7L2 mRNA will be the focus of our future studies.

Our previous study revealed that Wnt signaling was augmented in cetuximab-resistant CRC cells and tumors that progressed on cetuximab treatment due to repression of the five negative regulators (DKK1, DKK3, ZNRF3, RNF43 and APC2) of Wnt signaling by *MIR100HG*-derived miRNAs, miR-100 and miR-125b [7]. In the present study, we provide further evidence regarding the activation of Wnt signaling by finding that MIR100HG interacted with hnRNPA2B1 to enhance Wnt signaling through stabilization of TCF7L2 mRNA, with TCF7L2 as a core component that binds to nuclear β-catenin to promote downstream Wnt target gene transcription [46]. Based on our findings, we propose that MIR100HG and miR-100/125b function at different levels of Wnt signaling via complementary mechanisms that augment Wnt signaling activity in the setting of advanced CRC (Supplementary Fig. 5c). Our findings are in line with the recent studies supporting that lnc-MIRHGs frequently function in concert with their encoded miRNAs. For instance, miR503HG and its encoded miR-503 cooperatively inhibit tumor metastasis in hepatocellular carcinoma [47, 48], and lncRNA H19 and miR-675 both promote tumor cell proliferation in CRC [49, 50].

Wnt signaling is considered a potent regulator of EMT in diverse cancer types [51]. In this study, we observed that the MIR100HG/hnRNPA2B1/TCF7L2-mediated activation of Wnt signaling coincides with an EMT signature in CRC. Consistently, EMT-TFs including ZEB1 and Slug, which are known Wnt target genes, were significantly modulated upon MIR100HG and hnRNPA2B1 overexpression or knockdown. It is well acknowledged that EMT is a key driver of cancer metastasis [1], but EMT also can override the cytostatic effects of anti-EGFR therapy by increasing Snail and AXL expression [52]. In addition, EMT confers anti-EGFR therapy resistance by reducing BIM expression, which is transcriptionally repressed by the EMT-TF ZEB1 [53]. Based on the above evidence, we speculate that MIR100HG mainly functions via the regulation of EMT. MIR100HG overexpression appears to have proportionally greater effects on inducing mesenchymal genes than suppressing epithelial genes within the EMT program.

We cannot exclude that other Wnt target genes or signaling pathways may also contribute to the acquisition of drug resistance and metastatic capability. Notably, accumulating evidence indicates that activation of EMT via the Wnt signaling pathway is closely linked to cancer cells adopting a cancer stem cell (CSC) state [2, 54, 55]. In the present study, we did notice elevated expression of CD44, a CSC-associated cell-surface marker, after MIR100HG expression in CRC cells. Delving deeper into roles for and mechanism(s) by which MIR100HG achieves a CSC phenotype will be the focus of our future studies that may reveal additional dimensions to EMT in driving CRC progression.

m^6^A modifications play a pivotal role in controlling RNA fate [33]. Recently, it was reported that TCF7L2 mRNA is rich in m^6^A modifications and that these alterations can affect TCF7L2 expression [36]. Consistent with this, we found that the m^6^A modification of TCF7L2 mRNA is indispensable for its stabilization and interaction with hnRNPA2B1. Our results also support recent evidence that hnRNPA2B1 acts as a m^6^A reader and that the recognition of m^6^A sites might be a general mechanism mediating the binding between hnRNPA2B1 and its target RNA transcripts [26, 27]. Considering that m^6^A modifications are relevant to cancer development and catalyzed predominately by the METTL3-METTL14 methyltransferase complex, a selective METTL3 inhibitor has recently been developed and exhibits a significant therapeutic effect in acute myeloid leukemia [56]. In this study, we found that METTL3 interference dramatically hindered the interaction between TCF7L2 mRNA and hnRNPA2B1, thus leading to reduced TCF7L2 expression. Therefore, targeting METTL3 by small molecules may be a promising strategy to overcome TCF7L2 augmented cetuximab resistance in CRC, a direction which deserves further study.

Our findings have important therapeutic implications for targeting lncRNA MIR100HG in CRC patients. Recent studies support that targeting specific oncogenic lncRNAs by ASOs, like targeting TUG1 in glioma [57] or LINC02273 in breast cancer [58], have significant therapeutic value. Although challenging, it may be beneficial to develop MIR100HG-targeting therapies to treat cetuximab-resistant or metastatic CRC. More interestingly, EMT also plays a pivotal role in tumor immunosuppression and immune evasion, and the interplay between EMT and PD-L1 signaling contributes to therapeutic resistance of immune checkpoint blockade (ICB) [58, 59]. The use of nude mice excluded exploring the immunological effects of cetuximab treatment, which is a limitation of the current study. Therefore, combining a targeting strategy for MIR100HG with ICB might be a future treatment strategy to enhance patient responsiveness to immunotherapy in CRC.

## Conclusions

Our results uncover a previously unidentified role for MIR100HG in regulating EMT-related cetuximab resistance and metastasis in CRC by forming a regulatory circuit involving hnRNPA2B1 and TCF7L2. MIR100HG interacts with hnRNPA2B1 to mediate the stabilization of TCF7L2 mRNA in a m^6^A-dependent manner. TCF7L2, in turn, regulates MIR100HG transcription, closing the feed forward regulatory loop that leads to the increased activation of Wnt signaling.

## Supplementary Information

**Additional file 1.**

**Additional file 2.**

**Additional file 3.**

## Data Availability

All data that support the findings of this study are available from the corresponding authors upon reasonable request.

## Abbreviations

EMT: Epithelial-to-mesenchymal transition
lncRNA: Long non-coding RNA
CRC: Colorectal cancer
EGFR: Epidermal growth factor receptor
lnc-MIRHG: MiRNA-host gene lncRNA
m^6^A: N6-methyladenosine
hnRNPA2B1: Heterogeneous nuclear ribonucleoprotein A2B1
FFPE: Formalin-fixed paraffin-embedded
3D: Three-dimension
TF: Transcription factor
TCGA: The Cancer Genome Atlas
GSEA: Gene Set Enrichment Analysis
CMS: Consensus molecular subtyping
