# Supplementary material for: Interaction of lncRNA MIR100HG with hnRNPA2B1 facilitates m6A-dependent stabilization of TCF7L2 mRNA and colorectal cancer progression
Source: Mol Cancer. 2022 Mar 12;21:74. doi: 10.1186/s12943-022-01555-3 (PMC8917698; doi:10.1186/s12943-022-01555-3)
Supplement: Supplementary file 1 — Additional file 1. [file 12943_2022_1555_MOESM1_ESM.docx]

**Supplementary materials**

**Interaction of lncRNA MIR100HG with hnRNPA2B1 facilitates m^6^A-dependent stabilization of TCF7L2 mRNA and colorectal cancer progression**

Hao Liu, Danxiu Li, Lina Sun, Hongqiang Qin, Ahui Fan, Lingnan Meng, Ramona Graves-Deal, Sarah E. Glass, Jeffrey L. Franklin, Qi Liu, Jing Wang, Timothy J. Yeatman, Hao Guo, Hong Zong, Shuilin Jin, Zhiyu Chen, Ting Deng, Ying Fang, Cunxi Li, John Karijolich, James G. Patton, Xin Wang, Yongzhan Nie, Daiming Fan, Robert J. Coffey, Xiaodi Zhao, Yuanyuan Lu

**Supplementary Materials and Methods**

**Human CRC tissue samples**

Twelve pairs of CRC specimens pre- and post-cetuximab treatment were obtained from the Xijing Hospital of Digestive Diseases, Fudan University Shanghai Cancer Center, the First Affiliated Hospital of Zhengzhou University and Tianjin Medical University Cancer Institute and Hospital. Most pre-cetuximab treatment specimens were obtained from CRC patients who underwent radical resection, and their matched post-cetuximab treatment specimens were obtained at the time of disease progression after cetuximab treatment post recurrence. These samples were obtained by biopsy from metastatic sites; 9 of 12 metastatic sites were hepatic. All patients provided informed consent. The study was approved by the Ethics Committee of each hospital. The tissue microarrays containing samples from CRC and paired lymph node or distant metastases were purchased from Outdo Biotech (Shanghai, China). Formalin-fixed, paraffin-embedded (FFPE) tumor tissue sections were used for the RNAscope assay. The clinicopathological information of CRC patients included in this study is included in Supplementary Table 1, 2, 3 and 11.

**Constructs, oligonucleotides, cell infection and transfection**

The pcDNA3.1-based vectors encoding human full-length MIR100HG, MIR100HG-AS (antisense), hnRNPA2B1 and TCF7L2 were constructed as previously described [1]. The lentiviral vector containing the MIR100HG DNA sequence (LV-MIR100HG), MIR100HG-AS (LV-MIR100HG-AS), short hairpin sequence against MIR100HG (shMIR100HG) and negative control were constructed by GeneChem (Shanghai, China). siRNAs targeting hnRNPA2B1 and TCF7L2 and anti-sense nucleotides (ASO) specifically targeting MIR100HG were purchased from RiboBio (Guangzhou, China). Sequences of siRNAs, ASO and shRNA used in this study are listed in Supplementary Table 4.

Oligonucleotides and vectors were transfected into indicated cells using JET (jetPRIME) following the manufacturer’s instructions. To generate stable cell lines, the indicated cells were infected with lentiviruses at a multiplicity of infection of 20:1 in a solution containing 5 μg/ml polybrene and subjected to drug selection with 2 μg/ml puromycin for 1 week. The knockdown efficiency and specificity of all siRNAs, ASO and shRNAs were validated by either RT-qPCR or immunoblots.

**Protein extraction and western blot**

Proteins were harvested with RIPA buffer supplemented with protease inhibitor cocktail (Sigma-Aldrich) and phosphatase inhibitor (Roche). The nuclear and cytoplasmic extract was isolated using a cytoplasmic and nuclear protein extraction kit (Thermo Fisher Scientific) following the manufacturer’s instructions. Protein concentration was determined using a BCA Protein Assay Kit (Thermo Fisher Scientific). Approximately 20–50 μg of denatured protein was resolved by SDS-PAGE and transferred to nitrocellulose membranes. The antibodies used in this study are summarized in Supplementary Table 5. Proteins were visualized using Dura SuperSignal Substrate (Thermo Fisher Scientific). Blots were scanned using a Molecular Imager ChemiDox XRS+ Imaging System with Image Lab software (Bio-Rad Laboratories). Original images of immunoblots are presented in Supplementary Figure 6.

**RNA isolation and RT-qPCR**

Total RNA was extracted using a RNeasy Plus Mini Kit (Qiagen) according to the manufacturer’s instructions. cDNA was synthesized using a PrimeScript RT reagent kit (TaKaRa). SYBR Premix Ex Taq II (TaKaRa) was used to amplify the double-stranded cDNA of interest. RT-qPCR primers for the genes of interest were synthesized by TaKaRa. The levels of ACTB were used as internal controls. A standard curve was established by amplifying diluted cDNA samples for calculation of relative target concentrations using Express SYBR GreenER qPCR SuperMix with Premixed ROX (Life Technologies). The 2^–ΔΔCt^ method was used to determine the relative expression level of RNA between groups. The primer sequences used in this study are listed in Supplementary Table 6.

**Generation of knockout cell lines**

Human codon-optimized Cas9 (hCas9) and GFP-targeting guide RNA (gRNA)-expressing plasmids (gRNA_GFP-T1) were purchased from Addgene. Two pairs of gRNAs targeting the adjacent sequences of MIR100HG exon 4 were designed using the CRISPRdirect web server, and the GFP-targeting sequence in the gRNA vector was replaced using QuickChange site-directed mutagenesis. To construct the knockout cell lines, the gRNA-expressing plasmid, hCas9 plasmid and pEGFP-C1 vector were co-transfected into CC-CR and HCT116 cells. GFP-positive cells were sorted into single clones into a 96-well plate by flow cytometry. Single clones were screened by the T7 endonuclease I-cutting assay. Knockout clones were confirmed by DNA sequencing. Target sequences of gRNA are listed in Supplementary Table 7.

**Immunofluorescence**

Immunofluorescence and confocal analysis were performed as described [2]. Briefly, cells in 2D and 3D culture were fixed in 4% PFA for 30 min, then permeabilized with 0.5% Triton X-100 for 10 min, stained with appropriate antibodies, and followed by confocal microscopic analysis. Primary antibodies used in immunofluorescent staining are summarized in Supplementary Table 5. Secondary antibodies were Alexa Fluor 568-conjugated or 488-conjugated goat anti-rabbit or goat anti-mouse IgG antibody (Life Technologies). Hoechst was used for nuclear staining. Slides were mounted with mounting medium before imaging with a Nikon ECLIPSE Ti confocal microscope.

**Immunohistochemistry (IHC)**

IHC staining was conducted on sections from tumor tissues of nude mice xenografts and CRC patients. Briefly, tissue sections were deparaffinized, subjected to heat-mediated antigen retrieval with 10 mM sodium citrate buffer (pH 6.0) and endogenous peroxidase inactivation, and incubated with primary antibodies against cleaved caspase-3 (#9664, Cell Signaling Technology), Ki-67 (#9449, Cell Signaling Technology) and hnRNPA2B1 (sc-374053, Santa Cruz). Sections were incubated with a peroxidase conjugated secondary antibody (Santa Cruz), followed by visualization with diaminobenzidine and image acquisition with a light microscope (Olympus). IHC slides were independently scored by two independent observers. The immunostaining intensity was scored on a scale of 0 to 3: 0 (negative), 1 (weak), 2 (moderate) or 3 (strong). The percentage of positive cells was evaluated on a scale of 0 to 4: 0 (negative), 1 (1%–25%), 2 (26%–50%), 3 (51%–75%), or 4 (76%–100%). The final IHC scores were calculated by multiplying the above two scores, resulting in an overall score which ranges from 0~12. A final score ranging from 0~3 indicated a “negative” case and the final score ranging from 4~12 suggested a “positive” case.

**RNA stability assay**

Cells were seeded into 6-well plates to achieve 50% confluency after 24 h, followed by treatment with 5 μg/ml actinomycin D (Sigma-Aldrich) for the indicated times. Cells at the indicated time point were collected for RNA isolation and subsequent RT-qPCR. The half-life of mRNA was calculated as previously described [3].

**Transwell migration and invasion assay**

Migration and invasion capacity of indicated cells were assessed using Transwell assays. Briefly, transfected or infected cells were suspended in serum-free DMEM, and 1×10^5^ cells were plated in the top chamber of the Transwell with an uncoated 8.0 μm pore membrane (Corning, for migration assay) or coated with diluted Matrigel (Corning, for invasion assay). Medium supplemented with 20% fetal bovine serum was added to the lower chamber. After incubation in 5% CO_2_ at 37°C for 24 or 48 h, cells that had migrated or invaded through the membrane were stained with 0.1% crystal violet and then imaged and counted under a microscope (Olympus) at ×200 magnification over three random fields in each well. Each experiment was performed in triplicate.

***In vivo* drug sensitivity assay**

Approximately 6×10^6^ tumor cells in 150 μl PBS were subcutaneously injected into the flanks of 6~8-week-old female athymic BALB/c nude mice (n=7). Mice weight and tumor size were measured regularly. Tumor volume was calculated using the following formula: tumor maximum diameter (L) × the right-angle diameter to that axis (W)^2^/2. Mice were treated with cetuximab (1 mg/mouse, intraperitoneal (i.p.), every 3 days) when tumor size reached around 100 mm^3^. The IVIS Spectrum In Vivo Imaging System (PerkinElmer) was used to detect bioluminescent signal in tumor-bearing mice. After 10 rounds of treatment, the mice were sacrificed, and tumors were weighed and imaged. Paraffin sections were prepared, and IHC was performed for Ki-67 and cleaved caspase-3. Data collection and analysis were done by two investigators blinded to the group allocations. The protocol for the animal studies was approved by the Fourth Military Medical University Institutional Animal Care and Use Committee.

***In vivo* metastasis assay**

*In vivo* metastatic ability of indicated cells was determined by tail vein injection of the cells into 6~8-week-old female nude mice as previously described [4]. SW620 and LoVo cells were employed due to their effective metastasis rates [5]. Briefly, 5×10^6^ MIR100HG-silenced cells or scramble control cells transduced with a luciferase-expressing lentiviral vector were injected via the tail vein into nude mice. Six weeks after injection, D-luciferin (Xenogen) at 100 mg/kg was injected intraperitoneally into the mice at weekly intervals, and bioluminescence was detected using the IVIS Spectrum In Vivo Imaging System (PerkinElmer). Mouse survival was recorded daily. Eight weeks after injection, mice were sacrificed and examined for lung metastasis using standard histological examination. Animal studies were conducted under the protocol approved by the Fourth Military Medical University Institutional Animal Care and Use Committee.

**Luciferase reporter assay**

A wild-type 263-nt sequence containing the +2133 m^6^A site of TCF7L2 was synthesized and cloned into the XhoI site of a pMIR-REPORT vector (Ambion). Mutations of the predicted m^6^A modification site in TCF7L2 were generated by QuickChange site-directed mutagenesis. HEK293T or HCT8 cells were seeded in triplicate in 24-well plates and allowed to reach 70% confluency the following day. Co-transfections of Reporter plasmid (100 ng) and Renilla luciferase (Rluc) control plasmid (pRL-TK) (20 ng) with or without hnRNPA2B1 expression vectors, MIR100HG ASO (RiboBio) and METTL3 siRNA (RiboBio) were conducted using Lipofectamine 2000 (Invitrogen).

To measure promoter activities using a luciferase reporter assay, the WT, truncated and MUT constructs of MIR100HG promoter fragments were subcloned upstream of the fireﬂy luciferase reporter in a pGL3-Basic vector. HCT116 and CC-CR cells were co-transfected with pGL3-MIR100HG promoter fragments, a pRL-SV40 Renilla luciferase reporter and either TCF7L2 siRNA or control. The firefly and Renilla luciferase activities were measured using the Dual-Luciferase Reporter Assay System (Promega). Firefly luciferase activity was normalized to Renilla activity and was presented as the relative luciferase activity.

**RNA sequencing**

Total RNA from MIR100HG-silenced and hnRNPA2B1-silenced HCT116 cells and scramble control cells were isolated by TRIzol Reagent (Invitrogen) and purified using an RNeasy Mini Kit (Qiagen). RNA-Seq was performed as previously described [1].

**RNA immunoprecipitation (RIP)**

RIP assays were conducted using a Millipore EZ-Magna Nuclear RIP (Native) Kit (Millipore) according to the manufacturer’s instructions. Briefly, 5 μg of FLAG antibody (F3165, Sigma-Aldrich), hnRNPA2B1 (sc-374053, Santa Cruz) antibody or a positive control EZH2 antibody (CS203195, Millipore) and corresponding control IgG (CS200621, Millipore) were conjugated to protein A/G magnetic beads by incubation for 30 min at room temperature, followed by washing 3 times and incubation with pre-cleared nuclear extracts in RIP buffer. The retrieved RNA was subjected to RT-qPCR, using total RNA as an input control. For the RT-qPCR analysis, U1 was used as a negative control, and NEAT1 was used as a positive control. All RIP assays were performed for three biological replicates.

**MeRIP-qPCR**

m^6^A modification of an individual gene was determined using the Magna methylated RNA immunoprecipitation m^6^A Kit (Millipore) with reference to the manufacturer’s instructions. Briefly, 5 μg of anti-m^6^A antibody (CS220007, Millipore) or normal mouse IgG (CS200621, Millipore) was prewashed and incubated with Magna ChIP protein A/G magnetic Beads (CS203152, Millipore) for 30 min at room temperature, then mixed with purified poly-(A) RNA. Enrichment of m^6^A containing mRNA was then analyzed by RT-qPCR and primers to a m^6^A negative region and m^6^A positive region of EEF1A were used as a negative and positive control, respectively. The corresponding m^6^A enrichment in each sample was calculated by normalizing to the input. Primers used in MeRIP-qPCR are listed in Supplementary Table 6.

**RNA pull-down assay**

FL (full-length), AS (antisense) and serial truncations of MIR100HG RNA were transcribed with a HiScribe™ T7 Quick High Yield RNA Synthesis Kit (NEB) and purified with an RNeasy MinElute Cleanup Kit (QIAGEN), followed by labelling using an RNA 3’ End Desthiobiotinylation Kit (Thermo Fisher Scientific). Purified biotin-labeled RNA was heated and annealed to form a secondary structure, mixed with whole cell extract in RIP buffer for 1 h, and incubated with streptavidin agarose beads (Invitrogen) for 1 h. Finally, the RNA-binding proteins were analyzed by Western blot.

For *in vivo* S1m precipitation, we constructed S1m-tagged TCF7L2 as previously described [6]. Wild-type S1m-tagged TCF7L2 (TCF7L2-WT), mutant S1m-tagged TCF7L2 (TCF7L2-mut), or a S1m-vector was transfected into CC and HCT8 cells that were plated on 10 cm dishes. 48 h after transfection, the cells were harvested and lysed in 1.5 ml lysis buffer containing protein (Roche) and RNase inhibitor (Thermo Fisher Scientific). The supernatant was collected after 20 min of centrifugation at 4°C and incubated with streptavidin beads (Invitrogen) for 10 min at 4°C to remove background. The input consisted of 10% of the supernatant. The remaining lysate was incubated with streptavidin beads at 4°C for 4 h before washing 5 times with wash buffer. Finally, the pellet was resuspended in 40 μl of 2×SDS-PAGE sample buffer, followed by Western blot with a hnRNPA2B1 antibody.

**Chromatin immunoprecipitation (ChIP)**

ChIP assays were performed using a Pierce Agarose ChIP Kit (Thermo Fisher Scientific) following the manufacturer’s instructions. Rabbit anti-TCF7L2 antibody (#C48H11, Cell Signaling Technology) or normal rabbit IgG (#2729, Cell Signaling Technology) was used for immunoprecipitation. Recovered DNA was subjected to qPCR to amplify the binding sites of the MIR100HG promoter region. Relative enrichment was normalized to control IgG. The primers used are listed in Supplementary Table 6.

**Chromatin isolation by RNA purification (ChIRP)**

ChIRP assays were performed as described [7]. Briefly, 26 probes unique to the MIR100HG sequence and spanning the entire transcript were designed using the online designer at Stellaris (http://www.singlemoleculefish.com). Thirteen probes were designed against LacZ RNA as a negative control. Probes were labeled according to their positions along the RNA and separated into two pools so that the “even” pool contained all probes numbering 2, 4, 6, etc. and the “odd” pool contained probes numbering 1, 3, 5, etc. Experiments were performed using both pools, which served as internal controls for each other. All probes were synthesized to have a 3’ biotinylation modification. CC-CR and HCT116 cells overexpressing MIR100HG were grown to 80%~90% confluency in 200 mm dishes. Three dishes were used for each probe set. Cells were rinsed with 1× PBS twice and cross-linked with 1% glutaraldehyde (Sigma-Aldrich) for 10 min at room temperature then quenched with 10% volume of 1.25 M glycine at room temperature for 5 min. Cell pellets were collected and resuspended with nuclei lysis buffer. Lysates were sonicated in a 4°C water bath at the highest setting with 30 sec ON, 45 sec OFF pulse intervals until lysates were no longer turbid. Cell lysates were diluted in hybridization buffer with a probe concentration of 100 nM added and incubated by end-over-end rotation at 37°C for 4h. After incubation, pretreated magnetic streptavidin C1 beads were added to each hybridization reaction at a concentration of 100 μl per 100 pmol of probe followed by incubation at 37°C for 30 min by end-over-end rotation. Finally, DNA, RNA and protein were eluted and purified from the magnet beads for RT-qPCR or mass spectrometry and Western blot analyses. The sequences of the probes used in ChIRP assay are listed in Supplementary Table 8.

**TOP/FOP flash luciferase reporter assay**

TOP/FOP-Flash luciferase reporter assay was performed as previously described [8]. Briefly, the TOP/FOP-Flash reporter and pTK-RL plasmids were co-transfected into HEK293T cells with indicated treatment. Recombinant human Wnt3A (10 ng/ml) was used to activate the Wnt signaling. The firefly and *Renilla* luciferase activities were measured using the Dual-Luciferase Reporter Assay System (Promega). The TOP/FOP-Flash reporter activity was presented as the relative ratio of firefly luciferase activity to *Renilla* luciferase activity.

**Statistical analysis and data availability**

All analyses were performed using SPSS software (version 22.0). The data are presented as mean ± s.d.. Two-tailed unpaired or paired Student’s t test, ANOVA (Dunnett’s or LSD post hoc test), non-parametric signed rank test, Mann–Whitney U test, and Pearson correlation coefficients were used according to the type of experiment. P < 0.05 was considered significant.

**Supplementary Figure Legends**

**Supplementary Fig. 1 MIR100HG expression is closely related to EMT**

**(a)** qPCR analysis of EMT markers originating from 3D-cultured CC and CC-CR cells. n=3 independent biological replicates. **(b)** Correlation analyses of MIR100HG expression and expression of epithelial genes in TCGA database (n=433). **(c)** Left, schematic illustration of the targeting strategy to delete exon 4 of *MIR100HG* to generate *MIR100HG*^KOE4^ CC-CR and HCT116 cells by CRISPR/Cas9-mediated genome editing. Sequences targeted by CRISPR/Cas9 are shown in red. Protospacer adjacent motifs are shown in green. The sequence of the targeted region and two knockout alleles (KOE4#1 and KOE4#2) are shown. Right, representative agarose gel displaying PCR reactions with primers flanking exon 4 of *MIR100HG* using genomic DNA isolated from wild-type cells and two *MIR100HG*^KOE4^ clones. **(d)** qPCR analysis of MIR100HG, miR-100 and miR-125b expression after MIR100HG overexpression or silencing in indicated cells. n=3 independent biological replicates. **(e)** qPCR analysis of MIR100HG expression in CRC cells, fold-changes were normalized to CC. ****P* < 0.001, ***P* < 0.01, **P* < 0.05. Data represent mean ± s.d., n.s., not significant.

**Supplementary Fig. 2 MIR100HG drives cetuximab resistance and metastasis in CRC cells**

**(a)** Responsiveness to cetuximab in a panel of 29 CRC cell lines stratified by the gene expression–based consensus molecular subtyping (CMS). **(b)** Quantification of Ki-67 and cleaved caspase-3 positive cells in the indicated groups (n=5). **(c)** Representative IHC images and quantification of Ki-67 and cleaved caspase-3 from indicated xenografts (n=7) treated with CTX. **(d, e)** Representative mages of Transwell migration and invasion assays of indicated cells. **(f)** Incidence of lung metastasis of the indicated group. ***P* < 0.01, **P* < 0.05. Data represent mean ± s.d., n.s., not significant. CTX, cetuximab.

**Supplementary Fig. 3 MIR100HG specifically binds to hnRNPA2B1**

**(a)** Immunoblots of hnRNPA2B1 in indicated CRC cells, representative of three independent experiments. **(b)** Representative images of Transwell migration and invasion assays of HCT116 and LoVo cells transfected with siRNAs against hnRNPA2B1. **(c)** Representative images of Transwell migration and invasion assays of MIR100HG-overexpressing SW480 and DiFi cells transfected with siRNAs against hnRNPA2B1.

**Supplementary Fig. 4** **MIR100HG and hnRNPA2B1 regulate TCF7L2 expression and increase Wnt signaling**

**(a, b)** qPCR analyses of hnRNPA2B1 expression levels in indicated cells after MIR100HG overexpression (a) and silencing (b). n=3 independent biological replicates. **(c)** Immunoblots of hnRNPA2B1 in indicated cells after MIR100HG overexpression and silencing. Representative of three independent experiments. **(d, e)** Immunoblots of hnRNPA2B1 in the nucleus and cytoplasm of indicated cells after MIR100HG overexpression (d) and silencing (e). Representative of three independent experiments. **(f)** Immunoblots and qPCR analysis of TCF7L2 expression in MIR100HG-overexpressing SW480 cells and MIR100HG-silenced HCT116 cells. n=3 independent biological replicates. **(g)** Immunoblots and qPCR analysis of TCF7L2 in hnRNPA2B1-overexpressing SW480 cells and hnRNPA2B1-silenced HCT116 cells. n=3 independent biological replicates. **(h)** qPCR analysis of TCF7L2 pre-mRNA in indicated cells. n=3 independent biological replicates. **(i, j)** Assessment of TCF7L2 mRNA half-life (t_1/2_) in hnRNPA2B1- (i) and MIR100HG- (j) silenced HCT116 cells. n=3 independent biological replicates. **(k)** Representative images of Transwell migration and invasion assays of HCT116 cells with indicated treatment. **(l)** qPCR analysis of Wnt target genes in MIR100HG-overexpressing Caco-2 cells or MIR100HG-silenced HCT116 cells. n=3 independent biological replicates. **(m)** qPCR analysis of Wnt target genes in hnRNPA2B1-overexpressing HCT8 cells or hnRNPA2B1-silenced HCT116 cells. n=3 independent biological replicates. ***P* < 0.01, **P* < 0.05. Data represent mean ± s.d., n.s., not significant.

**Supplementary Fig. 5** **hnRNPA2B1 binds to TCF7L2 in an m^6^A-dependent manner**

**(a)** Schematic diagram of TCF7L2-WT and TCF7L2-mut firefly luciferase reporters. The 263-nt DNA sequence of WT TCF7L2 was inserted at the XhoI site ahead of the stop codon of the firefly luciferase gene in a pMIR-REPORT vector to give rise to the TCF7L2-WT reporter. For the TCF7L2-mut reporter, A-T substitutions (shown in red) were made within the m^6^A consensus site (in grey background). **(b)** Immunoblots of TCF7L2 in indicated CRC cells. Representative of three independent experiments. **(c)** Model of a new mode of MIR100HG and miR-100/125b in regulating Wnt signaling.

**Supplementary Table 1.** Clinic-pathological characteristics of metastatic CRC patients with paired specimen pre- and post-cetuximab treatment

| No.^1^ | Gender^2^ | Age | Primary site | Metastatic  sites | Differentiation^3^ | Cetuximab regimen^4^ | Best response to cetuximab^5^ | Site of specimen (pre) | Site of specimen (post) |
| --- | --- | --- | --- | --- | --- | --- | --- | --- | --- |
| 1 | M | 67 | left colon | liver | G2 | FOLFOX4+cetuximab | PR | sigmoid | liver lesion |
| 2 | M | 37 | rectum | liver | G2 | FOLFOX4+cetuximab | SD | liver lesion | liver lesion |
| 3 | M | 51 | left colon | lung, liver,  peritoneum | G2 | FOLFOX4+cetuximab | PR | sigmoid | liver lesion |
| 4 | M | 56 | rectum | liver, ureter | G2 | capecitabine+cetuximab | SD | rectum | liver lesion |
| 5 | M | 49 | left colon | liver | G2 | mFOLFOX6+cetuximab  capecitabine+cetuximab | PR | liver lesion | liver lesion |
| 6 | F | 52 | rectum | liver | G2 | FOLFIRI+cetuximab | PR | rectum | liver lesion |
| 7 | F | 56 | rectum | liver, lung | G2 | FOLFOX4+cetuximab | SD | rectum | liver lesion |
| 8 | M | 67 | rectum | liver, lung | G2 | FOLFOX4+cetuximab | SD | rectum | liver lesion |
| 9 | M | 58 | rectum | liver | G2 | FOLFOX4+cetuximab | PR | rectum | liver lesion |
| 10 | F | 81 | transverse  colon | liver, bone | G3 | cetuximab | SD | transverse  colon | transverse  colon |
| 11 | F | 39 | left colon | left ovary and  adnexa, lung | G1 | FOLFIRI+cetuximab | SD | sigmoid | left adnexal  mass |
| 12 | M | 52 | right colon | lung, liver,  peritoneal cavity | G1-2 | FOLFIRI+cetuximab | SD | hepatic  flexure of  colon | abdominal  wall mass |

1. Cases 1, 2, 3 in Fig. 7a denote subject Nos. 4, 6, and 10 in this table.
2. M, male, F, female.
3. G1, well-differentiated, G2, moderately differentiated, G3, poorly differentiated.
4. Chemotherapy regimens: FOLFOX4 or mFOLFOX6, modified FOLFOX6 (5-fluorouracil, leucovorin and oxaliplatin) or FOLFIRI (5-fluorouracil, leucovorin and irinotecan). Cetuximab 400 mg/m^2^ initial dose followed by 250 mg/m^2^ weekly thereafter with cetuximab dose intensity>90% were given to all subjects.
5. PR, partial response; SD, stable disease.

**Supplementary Table 2.** Clinical information of 14 paired primary CRC tissues, adjacent nontumor tissues and matched lymph node metastasis

| No.^1^ | Gender | Age | Location of cancer | Differentiation | AJCC stage |
| --- | --- | --- | --- | --- | --- |
| 1 | M | 48 | left colon | G3 | III |
| 2 | F | 75 | right colon | G2 | III |
| 3 | F | 54 | rectum | G1-2 | III |
| 4 | M | 45 | left colon | G2 | III |
| 5 | F | 57 | right colon | G2 | III |
| 6 | F | 43 | right colon | G3 | III |
| 7 | M | 70 | left colon | G2-3 | III |
| 8 | M | 51 | rectum | G3 | III |
| 9 | M | 56 | right colon | G2-3 | III |
| 10 | M | 57 | left colon | G2-3 | III |
| 11 | F | 68 | right colon | G2 | III |
| 12 | F | 37 | left colon | G3 | III |
| 13 | F | 80 | right colon | G3 | III |
| 14 | M | 52 | left colon | G3 | III |

1. Case 1 in Fig. 7d denotes subject No. 6 in this table.

**Supplementary Table 3.** Clinical information of 14 paired primary CRC tissues, adjacent nontumor tissue and matched distant metastasis

| No.^1^ | Gender | Age^2^ | Primary site | Metastatic sites | Differentiation |
| --- | --- | --- | --- | --- | --- |
| 1 | F | 53 | right colon | liver | G2 |
| 2 | F | NA | left colon | liver | G2 |
| 3 | M | 61 | left colon | liver | G2-3 |
| 4 | F | 66 | right colon | liver | G2-3 |
| 5 | M | 60 | left colon | liver | G2 |
| 6 | M | 73 | left colon | liver | G2 |
| 7 | M | 61 | right colon | liver | G2 |
| 8 | M | 47 | right colon | abdominal wall | G2 |
| 9 | M | 46 | rectum | liver | G2 |
| 10 | M | 67 | rectum | liver | G2 |
| 11 | M | NA | rectum | liver | G2 |
| 12 | M | 50 | rectum | chest wall | G2 |
| 13 | M | 59 | rectum | liver | G3 |
| 14 | M | 59 | rectum | liver | G2 |

1. Case 2 in Fig. 7d denotes subject No. 4 in this table.
2. NA, not available.

**Supplementary Table 4.** Oligonucleotides used in this study

| Oligonucleotide | Sequence |
| --- | --- |
| ASO-MIR100HG#1 | 5'-CAGACAGACAGAACCAAGAC-3' |
| ASO-MIR100HG#2 | 5'-TGGGCAGTAACCAGGGAGCT-3' |
| sihnRNPA2B1#1 | 5'-GAGGTGGTTATGACAACTA-3' |
| sihnRNPA2B1#2 | 5'-TGGTCATAATGCAGAAGTA-3' |
| siMETTL3#1 | 5'-GAGCCAGCCAAGAAATCAA-3' |
| siMETTL3#2 | 5'-CTGCAAGTATGTTCACTATGA-3' |
| siTCF7L2#1 | 5'-AGACGAGGGCGAACAGGAG-3' |
| siTCF7L2#2 | 5'-CGCCAACGACGAACTGATT-3' |
| shMIR100HG#1 | 5'-GCACTATAGATCATGAGCTT-3' |
| shMIR100HG#2 | 5'-TATGTATAGAGAGCTGCTGA-3' |
| shhnRNPA2B1#1 | 5'-GCTCTTTATTGGTGGCTTAAG-3' |
| shhnRNPA2B1#2 | 5'-GCTGTTTGTTGGCGGAATTAA-3' |

**Supplementary Table 5.** Antibodies used in this study

| Antibodies | Source | Catalog | Application^1^ | Dilution |
| --- | --- | --- | --- | --- |
| HnRNPA2B1 | Abcam | ab31645 | WB | 1:1000 |
|  | Santa Cruz | sc-374053 | IHC-P  RIP | 1:200  1:10 |
| TCF7L2 | Cell Signaling Technology | #2569 | WB  ChIP | 1:1000  1:50 |
| E-cadherin | Cell Signaling Technology | #14472 | WB  IF | 1:1000  1:50 |
| ZO-1 | Santa Cruz | sc-33725 | WB  IF | 1:200  1:50 |
| Vimentin | Cell Signaling Technology | #5741 | WB  IF | 1:1000  1:100 |
| N-cadherin | Cell Signaling Technology | #13116 | WB  IF | 1:1000  1:200 |
| Slug | Cell Signaling Technology | #9585 | WB | 1:1000 |
| ZEB1 | Cell Signaling Technology | #3396 | WB | 1:1000 |
| Cleaved Caspase-3 | Cell Signaling Technology | #9664 | IHC-P  IF | 1:2000  1:800 |
| Ki-67 | Cell Signaling Technology | #9449 | IHC-P  IF | 1:500  1:800 |
| METTL3 | Cell Signaling Technology | #86132 | WB | 1:1000 |
| Histone H3 | Cell Signaling Technology | #4499 | WB | 1:2000 |
| α-Tubulin | Cell Signaling Technology | #2125 | WB | 1:1000 |
| β-actin | Cell Signaling Technology | #4970 | WB | 1:1000 |
| Hoechst 33342 | Thermo Fisher Scientific | #62249 | IF | 1:5000 |

1. WB: Western Blot, IHC-P: Immunohistochemistry (Paraffin), ChIP Chromatin immunoprecipitation, IF: Immunofluorescence, RIP: RNA binding protein immunoprecipitation

**Supplementary Table 6.** PCR primers used in the study

| Primer name | Sequence |
| --- | --- |
| Primers for qPCR |  |
| ACTB sense | 5'-AGGTTTGGACTCACTTGACAGG-3' |
| ACTB antisense | 5'-AGCACTGTGTTGGCGTACAG-3' |
| MIR100HG sense | 5'-GTGGCAGAGTAAGGGATGGA-3' |
| MIR100HG antisense | 5'-GGGGATGAACCATTGACAAC-3' |
| hnRNPA2B1 sense | 5'-GGAGCTTTGTCCTAAGTCCTTG-3' |
| hnRNPA2B1 antisense | 5'-ATGTTCCTGCTACCACCAAAG-3' |
| TCF7L2 sense | 5'-TGGAGGGCTCTTTAAGGGG-3' |
| TCF7L2 antisense | 5'-GATCCGTTGGGGAGGTAGG-3' |
| E-cadherin sense | 5'-CCCGGGACAACGTTTATTAC-3' |
| E-cadherin antisense | 5'-GCTGGCTCAAGTCAAAGTCC-3' |
| Vimentin sense | 5'-TACAGGAAGCTGCTGGAAGG-3' |
| Vimentin antisense | 5'-ACCAGAGGGAGTGAATCCAG-3' |
| N-cadherin sense | 5'-CCTCCAGAGTTTACTGCCATGAC-3' |
| N-cadherin antisense | 5'-GTAGGATCTCCGCCACTGATTC-3' |
| Slug sense | 5'-ATCTGCGGCAAGGCGTTTTCCA-3' |
| Slug antisense | 5'-GAGCCCTCAGATTTGACCTGTC-3' |
| Twist1 sense | 5'-GCCAGGTACATCGACTTCCTCT-3' |
| Twist1 antisense | 5'-TCCATCCTCCAGACCGAGAAGG-3' |
| Zeb1 sense | 5'-AGGGCACACCAGAAGCCAG-3' |
| Zeb1 antisense | 5'-GAGGTAAAGCGTTTATAGCCTCTATCA-3' |
| Zeb2 sense | 5'-AAGCCAGGGACAGATCAGC-3' |
| Zeb2 antisense | 5'-GCCACACTCTGTGCATTTGA-3' |
| U1 sense | 5'-GGGAGATACCATGATCACGAAGGT-3' |
| U1 antisense | 5'-CCACAAATTATGCAGTCGAGTTTCCC-3' |
| c-Myc sense | 5'-GGGCTTCTCAGAGGCTTGG-3' |
| c-Myc antisense | 5'-GTCCTTGCTCGGGTGTTGTA-3' |
| Cyclin D1 sense | 5'-AGAGGCGGAGGAGAACAAAC-3' |
| Cyclin D1 antisense | 5'-GGCGGATTGGAAATGAACTT-3' |
| CD44 sense | 5'-CCAGAAGGAACAGTGGTTTGGC-3' |
| CD44 antisense | 5'-ACTGTCCTCTGGGCTTGGTGTT-3' |
| MMP7 sense | 5'-TCGGAGGAGATGCTCACTTCGA-3' |
| MMP7 antisense | 5'-GGATCAGAGGAATGTCCCATACC-3' |
| NKD1 sense | 5'-TGCCTCCTGAGAAGACTGAC-3' |
| NKD1 antisense | 5'-CATAGATGGTGTGCAGCAAG-3' |
| PROX1 sense | 5'-TCACCTTATTCGGGAAGTGC-3' |
| PROX1 antisense | 5'-GTACTGGTGACCCCATCGTT-3' |
| TCF7L2 primary transcripts sense | 5'-TGGCAAATGTTGCTGAAAGGG-3' |
| TCF7L2 primary transcripts antisense | 5'-AGGGTTACAAGTTTTTGCAATGGT-3' |
| Primers for m^6^A RIP qPCR | |
| TCF7L2 (-426) sense | 5'-GCTCCCAGACTACTCCGTTCC-3' |
| TCF7L2 (-426) antisense | 5'-GGGAAGCCGAAGATACAGGAG-3' |
| TCF7L2 (-118) sense | 5'-ACCTTGGACTCGTCTTTTTCTTG-3' |
| TCF7L2 (-118) antisense | 5'-TTTTCACCCACCAGCAGCAA-3' |
| TCF7L2 (+2133) sense | 5'-CCATTCTTATTTCAATTTCTCCTT-3' |
| TCF7L2 (+2133) antisense | 5'-ATTGGTTCGCAAGCTCGTATT-3' |
| TCF7L2 (+2699) sense | 5'-GAAACCCAGATGTCACCAAAT-3' |
| TCF7L2 (+2699) antisense | 5'-TGCTCAGACAGTGTCGCTAAAA-3' |
| TCF7L2 (+2884) sense | 5'-CAGTGGGAACCATCTTCGTTT-3' |
| TCF7L2 (+2884) antisense | 5'-TTGGCACGTAAAGTTTTGTACAC-3' |
| Primers for MIR100HG promoter construct | |
| (-2000/+500) MIR100HG | 5'-CGACGCGTGTCCCTGATGGGGACACGTATGCTCCAG-3' |
| (-1769/+500) MIR100HG | 5'-CGACGCGTAGTTTGTAACAGGGCTTAATTATCTT-3' |
| (-1717/+500) MIR100HG | 5'-CGACGCGTAGCCCTAGGCCCAACATAGGTCTGAG-3' |
| (+69/+500) MIR100HG | 5'-CGACGCGTGTTTCAAGATCCACGAGGAACAAGTC-3' |
| (+262/+500) MIR100HG | 5'-CGACGCGTTTTCTCTTTCCATTGTTCAAATATGT-3' |
| Antisense | 5'-CCGCTCGAGGAAGGGCTTTAGGTGTCACGATATCT-3' |
| Primers for MIR100HG promoter site-directed mutagenesis | |
| MIR100HG mut #1 sense | 5'-GTTTGTCCCCCCCGGTCCAAGTTTGTAACAGGGCTTAATTATCTTCAGGGCAGTTT-3' |
| MIR100HG mut #1 antisense | 5'-GCCCTGTTACAAACTTGGACCGGGGGGGACAAACAAAAACAAAAAACAAAACAAAA-3' |
| MIR100HG mut #2 sense | 5'-GGGCAGTTTAGTTTCCCTGCGCCTAGCCCTAGGCCCAACATAGGTCTGAGAAATAT-3' |
| MIR100HG mut #2 antisense | 5'-AGGGCTAGGCGCAGGGAAACTAAACTGCCCTGAAGATAATTAAGCCCTGTTACAAA-3' |
| Primers for ChIP in the MIR100HG promoter | |
| Bing site 1/2 sense | 5'-CTCTTATAATTTATTTGTTTTCACT-3' |
| Bing site 1/2 antisense | 5'-GGAAAGAGAAAAAAAATCAAAAGAT-3' |
| Bing site 3/4 sense | 5'-GCTGCTTTTCTTGATTTCTCTAAAT-3' |
| Bing site 3/4 antisense | 5'-CTATGAACCATAAACTCACTAAATA-3' |
| Distal sense | 5'-GGAGATTGACCACCAGAGACCCTCT-3' |
| Distal antisense | 5'-GGCATTAACTCATGGCTAGTAAGGC-3' |

**Supplementary Table 7.** gRNA sequences used in this study

| No. | Target gene | Target sequence |
| --- | --- | --- |
| gRNA#1 | *MIR100HG* | 5'-TGTGTGAGACACCACTCTAA-3' |
| gRNA#2 | *MIR100HG* | 5'-ATCTCTAGTGAGACTTAGCA-3' |
| gRNA#3 | *MIR100HG* | 5'-AGTTAGTTTCAAGATCCACG-3' |
| gRNA#4 | *MIR100HG* | 5'-ATTAGCTGACTTGTTCCTCG-3' |

**Supplementary Table 8.** ChIRP probes used in this study

| ChIRP probes | Sequence |
| --- | --- |
| PROBE-MIR100HG-1 | 5'-TCTGCGCTTCCAAAATTCCG-3' |
| PROBE-MIR100HG-2 | 5'-ATCTTAGACCCCATCATTGC-3' |
| PROBE-MIR100HG-3 | 5'-GTTACTATTCCTCCTTAATT-3' |
| PROBE-MIR100HG-4 | 5'-TCAGATGTCTTGGTTCTGTC-3' |
| PROBE-MIR100HG-5 | 5'-GAGGAGCAGAAGCGAGGAAG-3' |
| PROBE-MIR100HG-6 | 5'-GTACTATAGGACCAGCTGAA-3' |
| PROBE-MIR100HG-7 | 5'-TCATAGTTGGCTTGAGAGTC-3' |
| PROBE-MIR100HG-8 | 5'-AGAGTTCTAGTGGCAGAAGC-3' |
| PROBE-MIR100HG-9 | 5'-AGGGAAAGTTCTTGGCTCAT-3' |
| PROBE-MIR100HG-10 | 5'-GTGCAATTTGGGATTTTGAA-3' |
| PROBE-MIR100HG-11 | 5'-TCTGTAGGTCACTCTCAAGA-3' |
| PROBE-MIR100HG-12 | 5'-GTTAGTGTCAAGTGGACCAA-3' |
| PROBE-MIR100HG-13 | 5'-CTTATCCTCTACTGACTCTA-3' |
| PROBE-MIR100HG-14 | 5'-AGAGAGCTGCTGAATGTCTG-3' |
| PROBE-MIR100HG-15 | 5'-TCTCCATATTGTCACCAATC-3' |
| PROBE-MIR100HG-16 | 5'-GCTAATTGTTTGCCATGGAA-3' |
| PROBE-MIR100HG-17 | 5'-CTGATAAACTTCATTCCCCA-3' |
| PROBE-MIR100HG-18 | 5'-CCTTTTTTATTCTCAAGGAT-3' |
| PROBE-MIR100HG-19 | 5'-GACTGTTTCCACTCATCAAA-3' |
| PROBE-MIR100HG-20 | 5'-TACATTGAGGTGGGAAACCA-3' |
| PROBE-MIR100HG-21 | 5'-CCATTGAAGACATCATACAC-3' |
| PROBE-MIR100HG-22 | 5'-CATTCGTGCAGAAGCACTTA-3' |
| PROBE-MIR100HG-23 | 5'-TTCTGTGGCTGAAGTATTTT-3' |
| PROBE-MIR100HG-24 | 5'-TGTGTCTCAAACCTCTGTTA-3' |
| PROBE-MIR100HG-25 | 5'-GCCATGAGGAGATTGAAGTC-3' |
| PROBE-MIR100HG-26 | 5'-AAGTCACACTTTGTGCTTGG-3' |
| PROBE-LACZ-1 | 5'-GTGAATCCGTAATCATGGTC-3' |
| PROBE-LACZ-2 | 5'-TGAGGGGACGACGACAGTAT-3' |
| PROBE-LACZ-3 | 5'-TTCAGACGGCAAACGACTGT-3' |
| PROBE-LACZ-4 | 5'-CCCTGCCATAAAGAAACTGT-3' |
| PROBE-LACZ-5 | 5'-AGCAGCAGACCATTTTCAAT-3' |
| PROBE-LACZ-6 | 5'-GATCATCGGTCAGACGATTC-3' |
| PROBE-LACZ-7 | 5'-AAGCCATTTTTTGATGGACC-3' |
| PROBE-LACZ-8 | 5'-CGTTCATACAGAACTGGCGA-3' |
| PROBE-LACZ-9 | 5'-GTAGTTCAGGCAGTTCAATC-3' |
| PROBE-LACZ-10 | 5'-AAAGAAAGCCTGACTGGCGG-3' |
| PROBE-LACZ-11 | 5'-AATAAGGTTTTCCCCTGATG-3' |
| PROBE-LACZ-12 | 5'-GGAAGACGTACGGGGTATAC-3' |
| PROBE-LACZ-13 | 5'-ACACCAGACCAACTGGTAAT-3' |

**Supplementary Table 9.** Molecular subtypes of 29 CRC cell lines used in Figure S2a and their responsiveness to cetuximab

| CRC cell lines | CMS group^1^ | Mean CTX  inhibition rate (%)^2^ | CTX response  category |
| --- | --- | --- | --- |
| NCI-H508 | CMS2 | 83.4 | sensitive |
| V9P | CMS2 | 82 | sensitive |
| DiFi | CMS2 | 80.8 | sensitive |
| LIM1215 | CMS1 | 79.6 | sensitive |
| GEO | CMS2 | 68.7 | sensitive |
| SW403 | CMS2 | 66 | sensitive |
| SNUC4 | CMS3 | 48.3 | partially responsive |
| Caco2 | CMS4 | 47.7 | partially responsive |
| SW948 | CMS3 | 42.7 | partially responsive |
| HT29 | CMS3 | 36.5 | partially responsive |
| SK-CO-1 | CMS3 | 33.9 | partially responsive |
| DLD-1 | CMS1 | 24.9 | resistant |
| SW480 | CMS4 | 23.7 | resistant |
| SW837 | CMS4 | 21.8 | resistant |
| SW48 | CMS1 | 21.8 | resistant |
| SW620 | CMS4 | 14.5 | resistant |
| LoVo | CMS1 | 14.3 | resistant |
| COLO205 | CMS1 | 12.5 | resistant |
| T84 | CMS2 | 11.2 | resistant |
| LS174T | CMS3 | 9.7 | resistant |
| NCI-H716 | CMS4 | 9.7 | resistant |
| HCT8 | CMS1 | 8.4 | resistant |
| HCT15 | CMS1 | 4.5 | resistant |
| SW1116 | CMS2 | 2 | resistant |
| LIM2405 | CMS1 | 2 | resistant |
| RKO | CMS4 | 0.4 | resistant |
| LS123 | CMS4 | -4.8 | resistant |
| HuTu80 | CMS4 | -5.4 | resistant |
| HCT116 | CMS4 | -14.1 | resistant |

1. Data from Sveen, A., et al, *Clin Cancer Res*, 2018 [9].
2. Data from Jhawer, M. et al, Cancer Res, 2008 [10], and Lu, Y., et al, Nat Med, 2017 [1].

**Supplementary Table 11.** *KRAS, NRAS,* and *BRAF* mutational status and other mutations and amplifications status in CRC patients with paired specimens obtained prior to cetuximab (pre) and at time of tumor progression (post)

| No.^1^ | Combined analysis of  *KRAS, NRAS*, and *BRAF*^2^ | | Other mutations and  Amplifications |
| --- | --- | --- | --- |
|  | Pre | Post |  |
| 1 | WT | WT | SRC amplification |
| 2 | WT | WT | ND |
| 3 | WT | WT | PIK3CA (p.H1047TR) mutation |
| 4 | WT | NRAS p.Gln61Lys | ND |
| 5 | WT | WT | NA |
| 6 | WT | WT | ND |
| 7 | WT | WT | ND |
| 8 | WT | WT | ND |
| 9 | WT | WT | NA |
| 10 | WT | WT | ND |
| 11 | WT | NRAS c.182A>T p.Q61L | ND |
| 12 | WT | WT | ND |

1. Cases 1, 2, 3 in Fig. 7d denote subject No. 4, 6, and 10 in this table.
2. WT, wild-type, ND, not detected, NA, not available.

**Reference**

1. Lu Y, Zhao X, Liu Q, Li C, Graves-Deal R, Cao Z, et al. lncRNA MIR100HG-derived miR-100 and miR-125b mediate cetuximab resistance via Wnt/beta-catenin signaling. Nature medicine. 2017;23(11):1331-41.

2. Li C, Ma H, Wang Y, Cao Z, Graves-Deal R, Powell AE, et al. Excess PLAC8 promotes an unconventional ERK2-dependent EMT in colon cancer. The Journal of clinical investigation. 2014;124(5):2172-87.

3. Huang H, Weng H, Sun W, Qin X, Shi H, Wu H, et al. Recognition of RNA N(6)-methyladenosine by IGF2BP proteins enhances mRNA stability and translation. Nature cell biology. 2018;20(3):285-95.

4. Liu H, Du F, Sun L, Wu Q, Wu J, Tong M, et al. GATA6 suppresses migration and metastasis by regulating the miR-520b/CREB1 axis in gastric cancer. Cell death & disease. 2019;10(2):35-.

5. Wong CC, Qian Y, Li X, Xu J, Kang W, Tong JH, et al. SLC25A22 Promotes Proliferation and Survival of Colorectal Cancer Cells With KRAS Mutations and Xenograft Tumor Progression in Mice via Intracellular Synthesis of Aspartate. Gastroenterology. 2016;151(5):945-60.e6.

6. Hu X, Peng WX, Zhou H, Jiang J, Zhou X, Huang D, et al. IGF2BP2 regulates DANCR by serving as an N6-methyladenosine reader. Cell death and differentiation. 2020;27(6):1782-94.

7. Chu C, Quinn J, Chang HY. Chromatin isolation by RNA purification (ChIRP). Journal of visualized experiments : JoVE. 2012(61).

8. Hsu W, Liu L, Chen X, Zhang Y, Zhu W. LncRNA CASC11 promotes the cervical cancer progression by activating Wnt/beta-catenin signaling pathway. Biological research. 2019;52(1):33.

9. Sveen A, Bruun J, Eide PW, Eilertsen IA, Ramirez L, Murumägi A, et al. Colorectal Cancer Consensus Molecular Subtypes Translated to Preclinical Models Uncover Potentially Targetable Cancer Cell Dependencies. Clinical cancer research : an official journal of the American Association for Cancer Research. 2018;24(4):794-806.

10. Jhawer M, Goel S, Wilson AJ, Montagna C, Ling YH, Byun DS, et al. PIK3CA mutation/PTEN expression status predicts response of colon cancer cells to the epidermal growth factor receptor inhibitor cetuximab. Cancer research. 2008;68(6):1953-61.
