# Supplementary material for: Interaction of lncRNA MIR100HG with hnRNPA2B1 facilitates m6A-dependent stabilization of TCF7L2 mRNA and colorectal cancer progression
Source: Mol Cancer. 2022 Mar 12;21:74. doi: 10.1186/s12943-022-01555-3 (PMC8917698; doi:10.1186/s12943-022-01555-3)

Supplementary figure 1

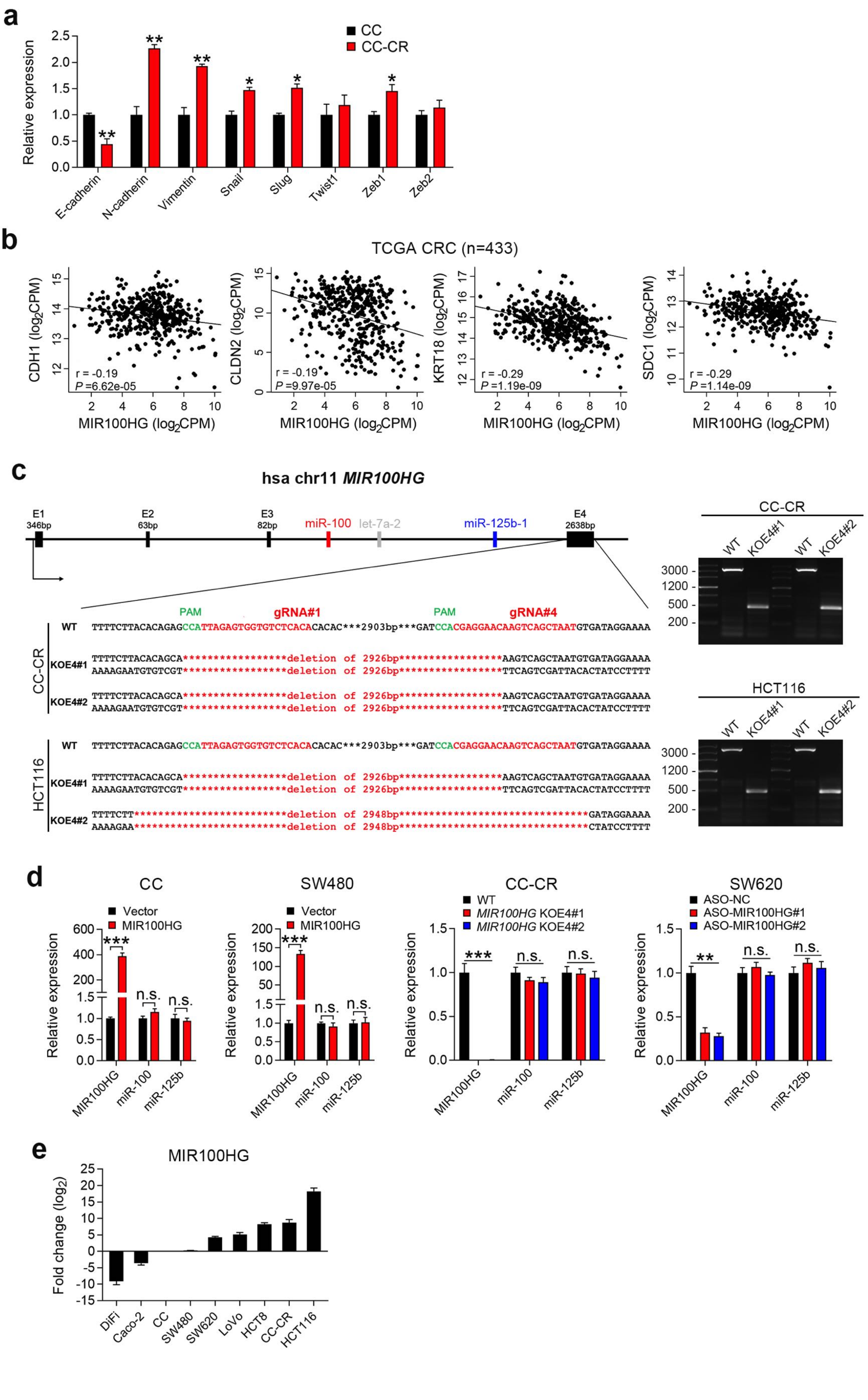

Supplementary figure 2

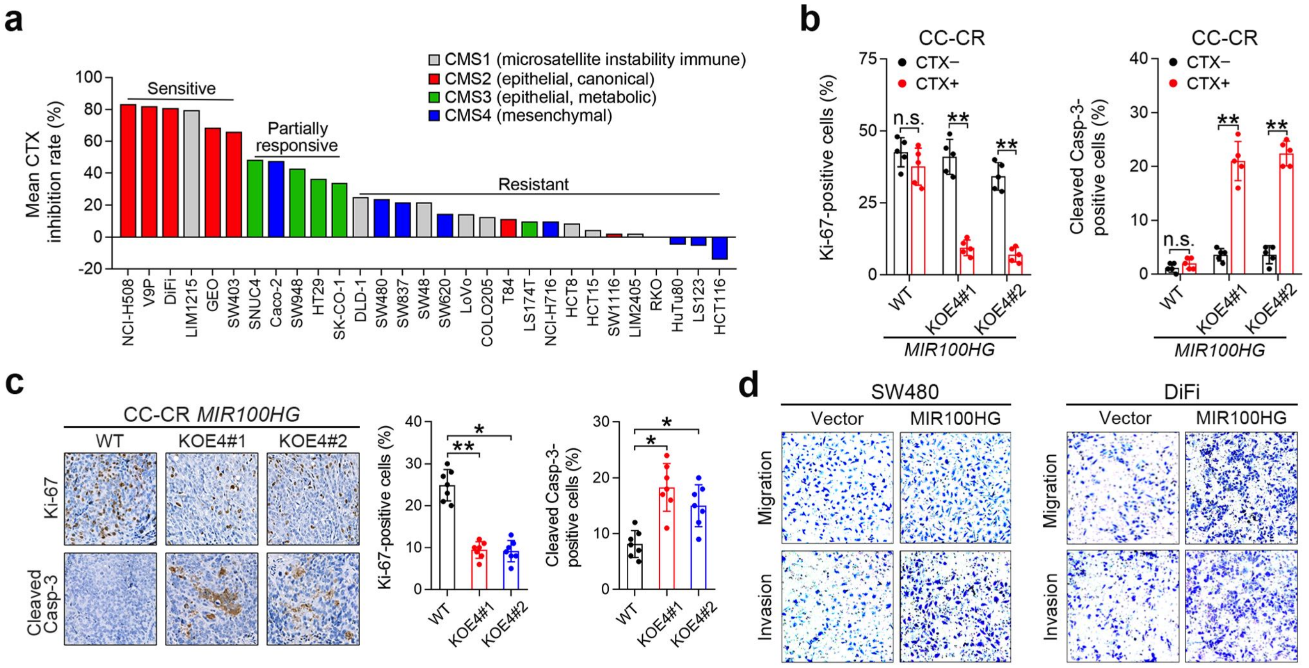

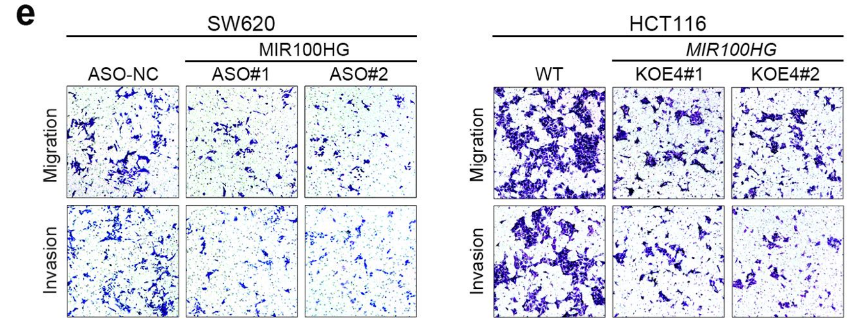

f

Incidence of lung metastasis in transplanted nude mice

	metastasis
SW620 shNC	8/10
SW620 shMIR100HG#1	2/10
SW620 shMIR100HG #2	3/10
LoVo shNC	9/10
LoVo shMIR100HG#1	3/10
LoVo shMIR100HG#2	4/10

Supplementary figure 3

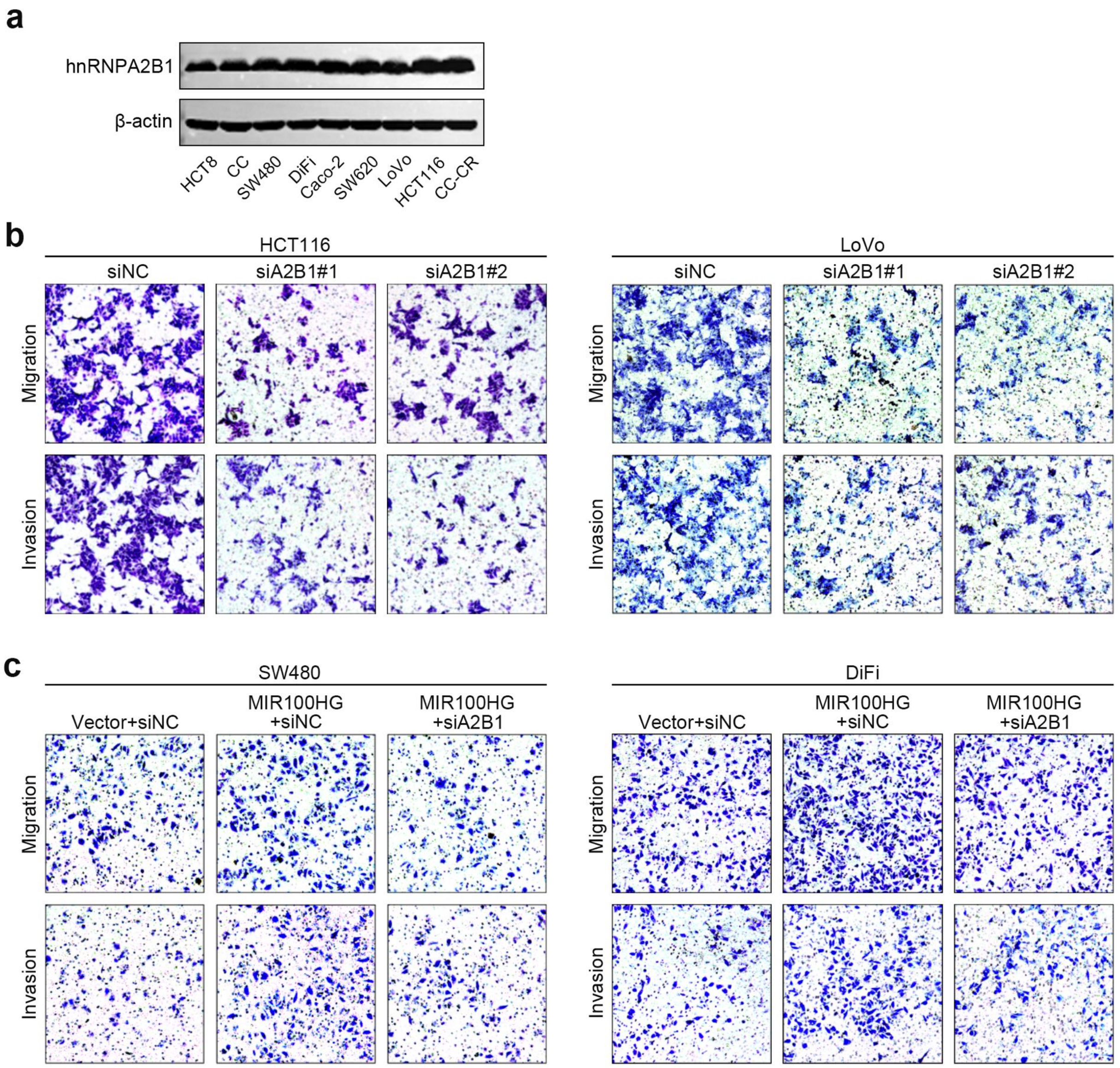

Supplementary figure 4

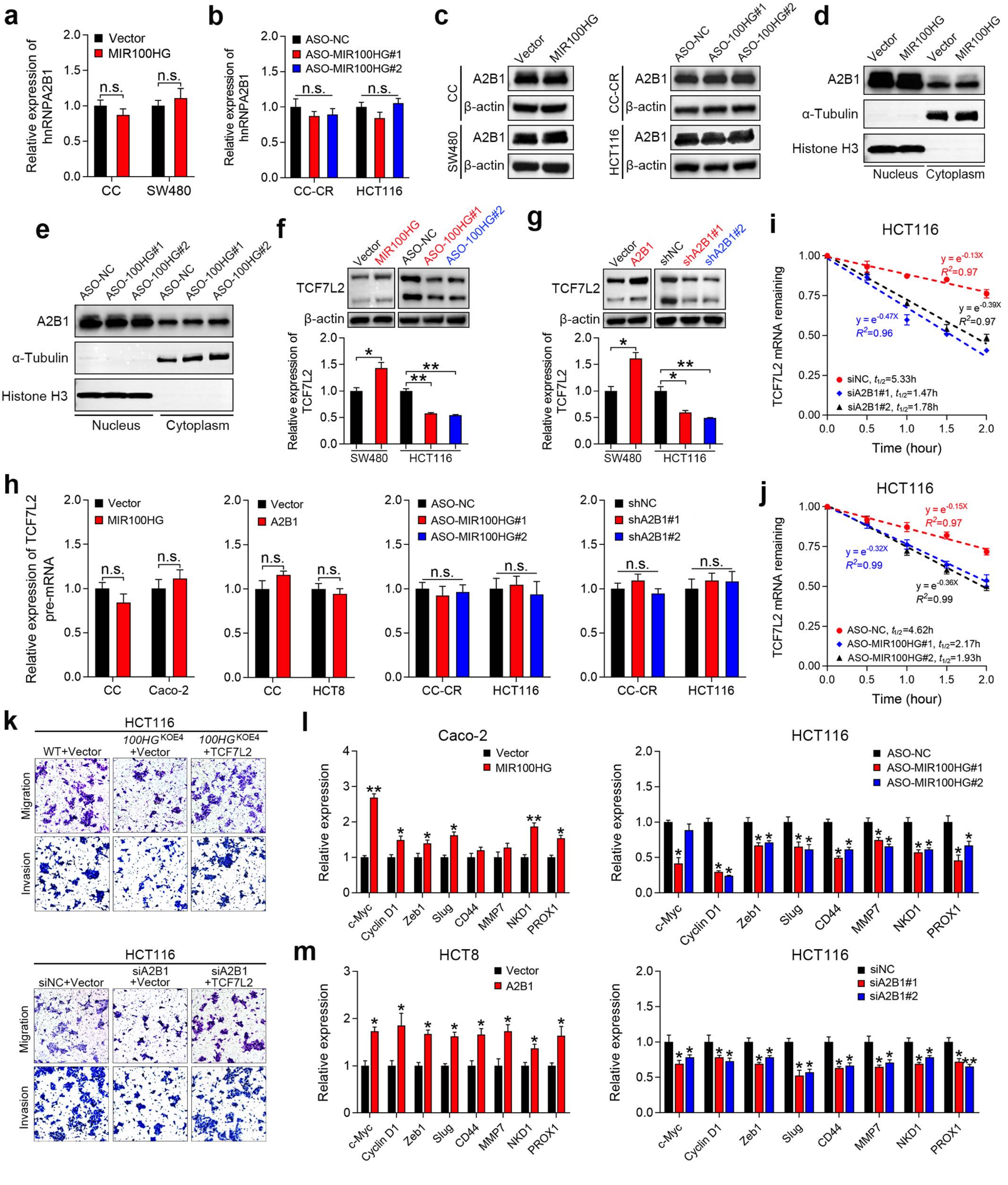

Supplementary figure 5

a

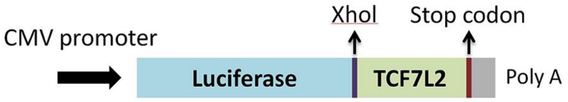

TCF7L2-WT (2482-2744)：

5′ ...aactgttatgtagttcggatagcttagttttaaaagactgattaaaaaacaaaaagaaaaaaaaagc... 3′

TCF7L2-mut：

5′ ...aactgttatgtagttcggatagcttagttttaaaagtctgattaaaaaacaaaaagaaaaaaaaagc... 3′

b

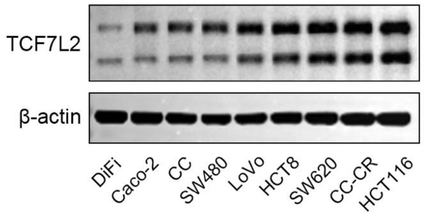

c

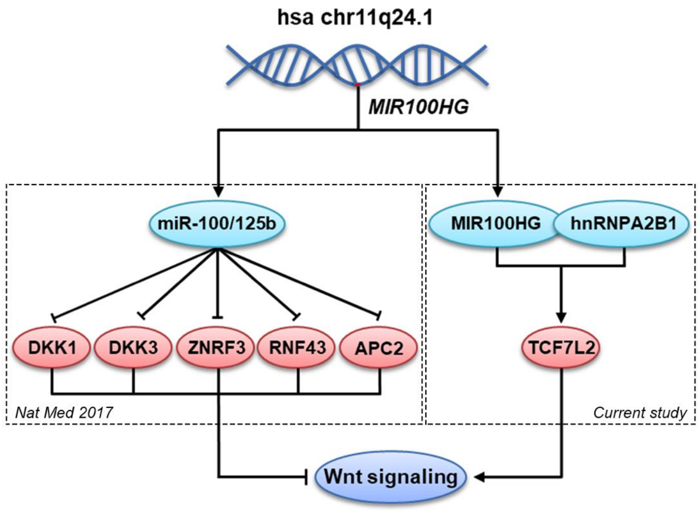

Supplementary figure 6

Figure 1c

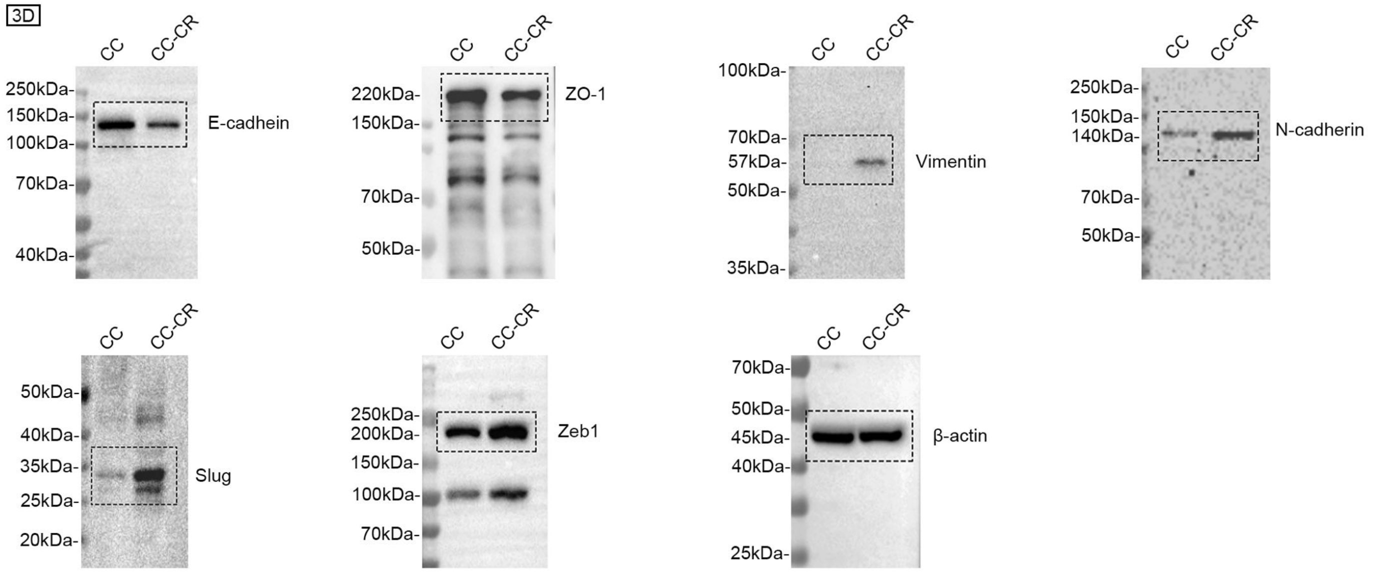

Figure 1h

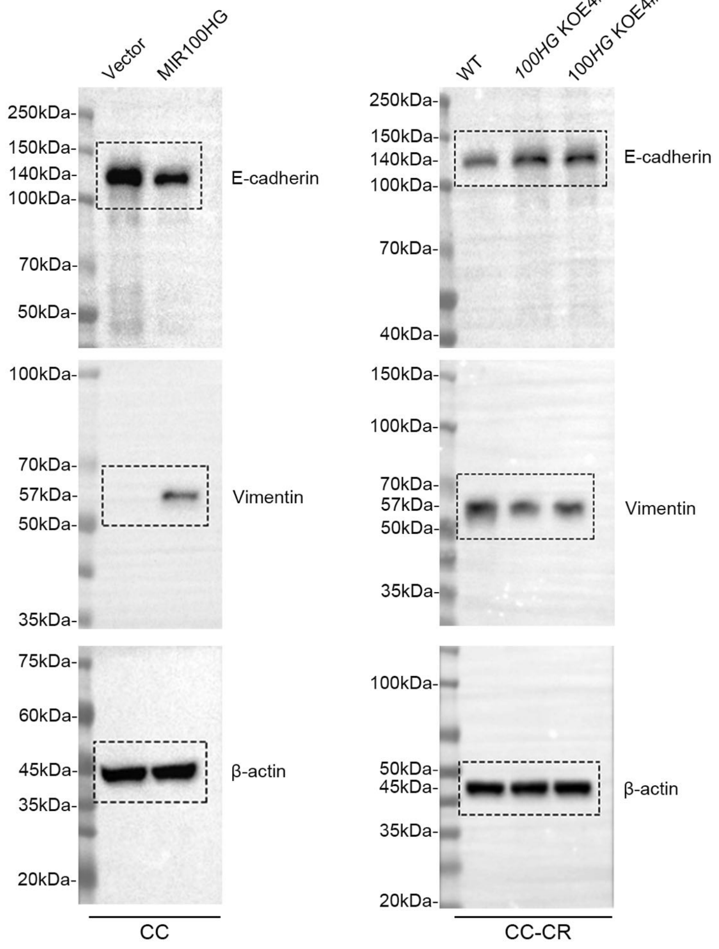

Figure 1j

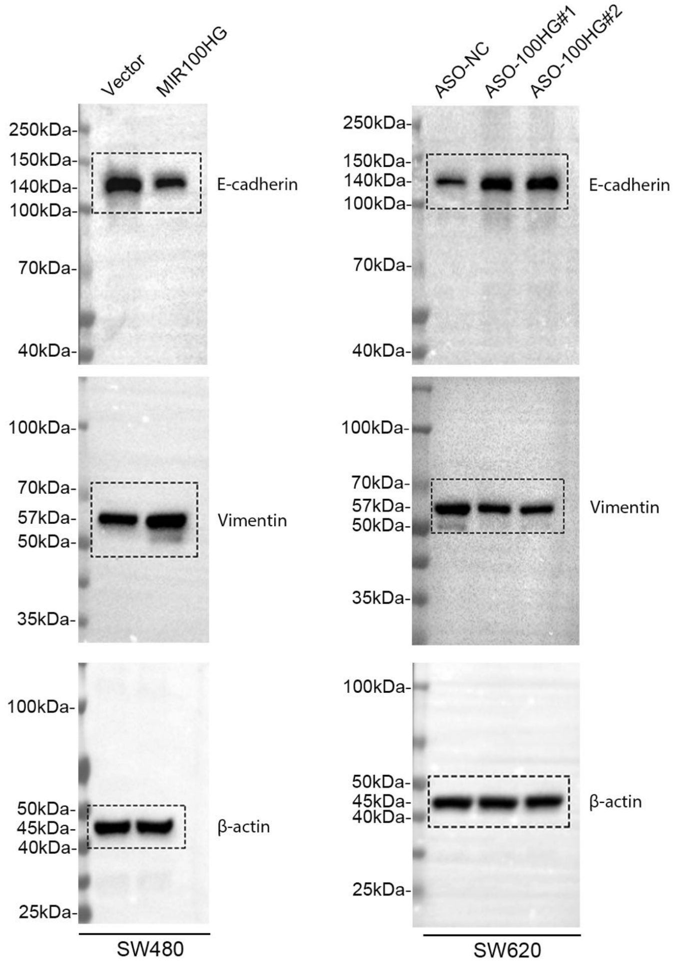

Figure 3a

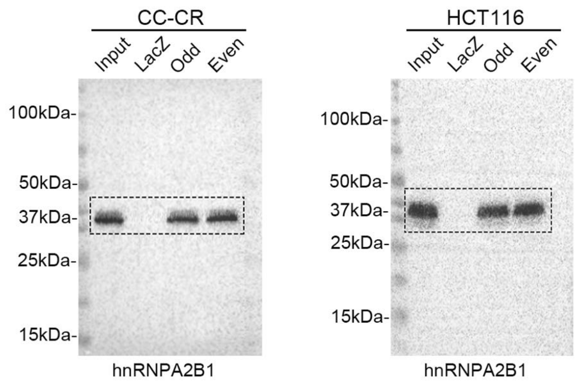

Figure 3b

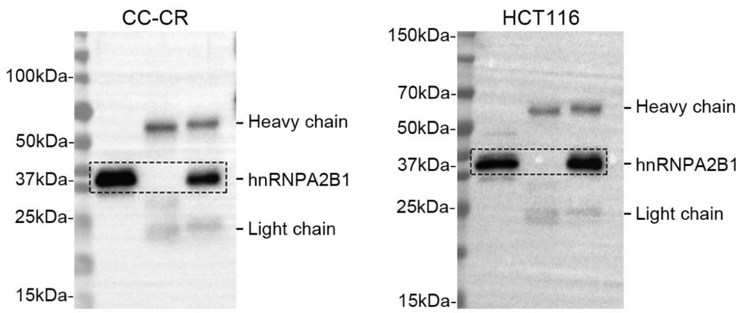

Figure 3d

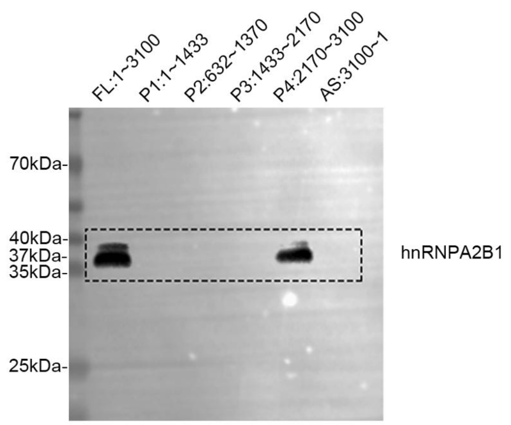

Figure 3g

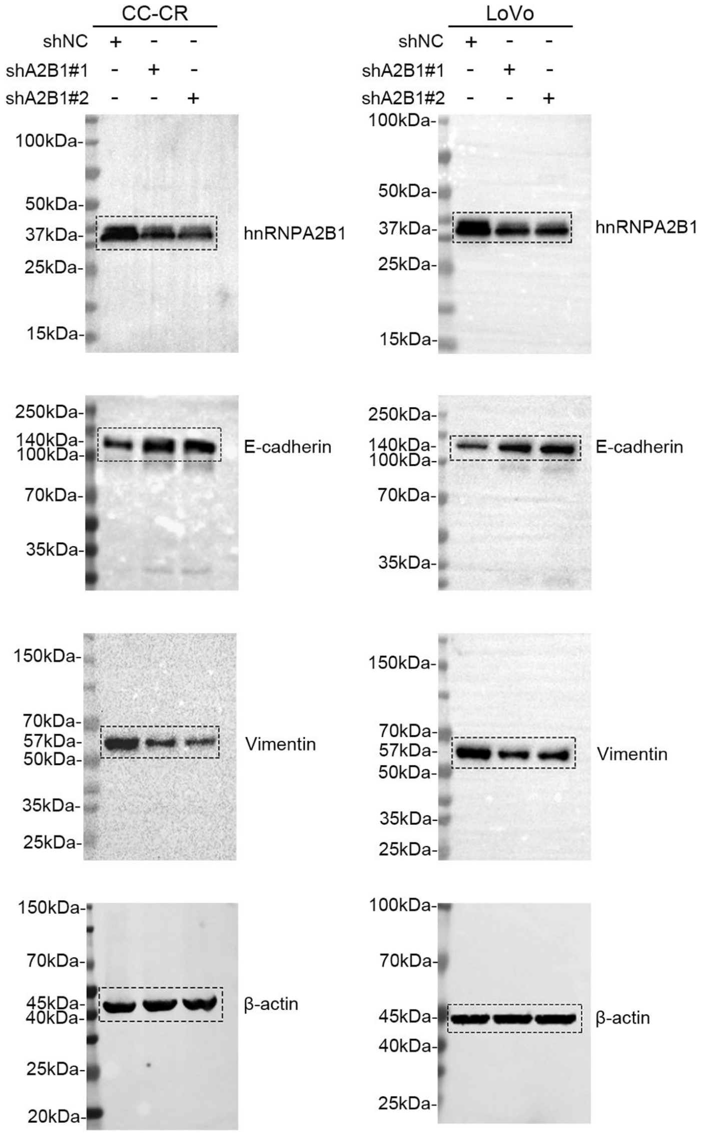

Figure 3j

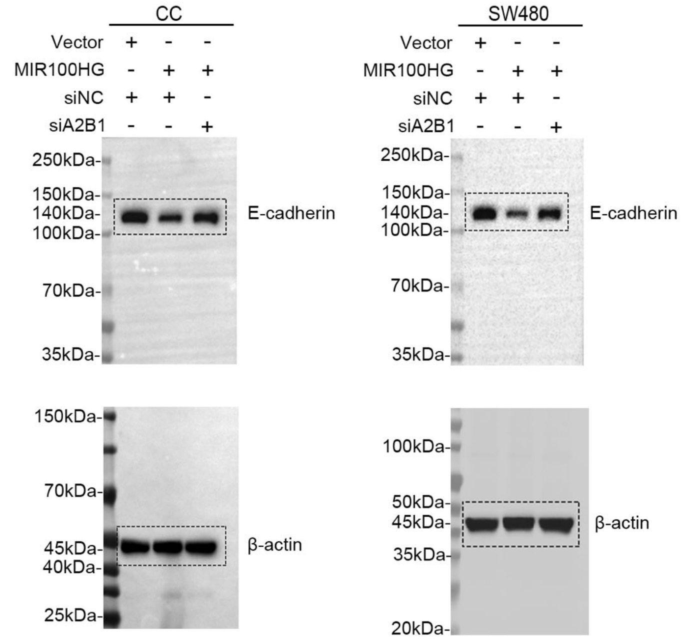

Figure 4b

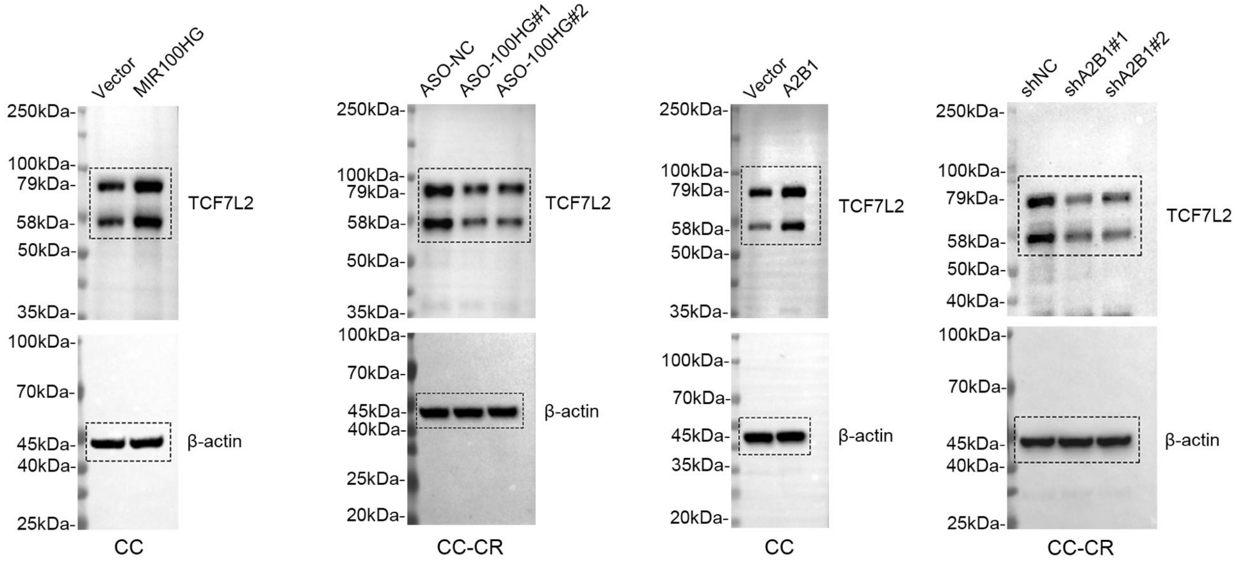

Figure 4h

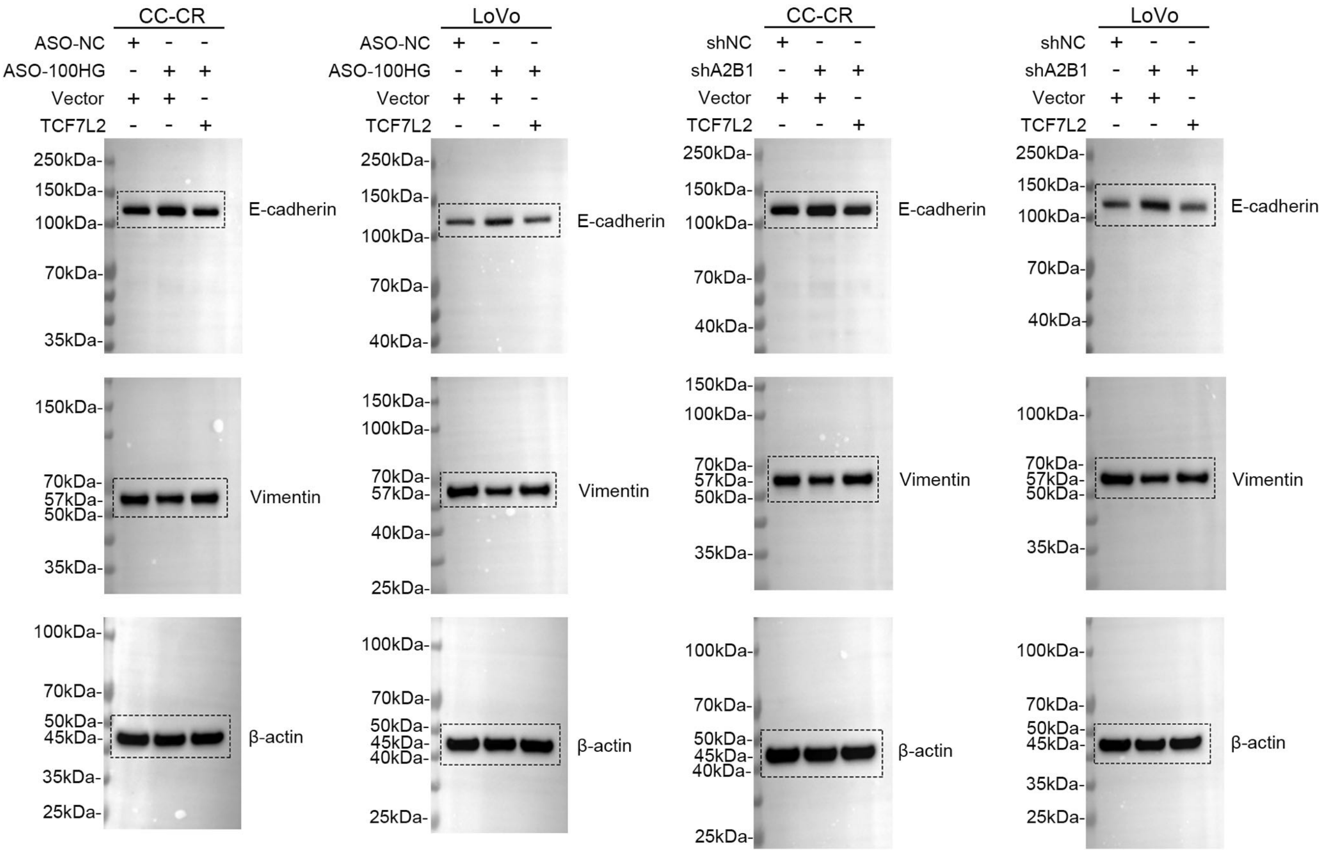

Figure 5a

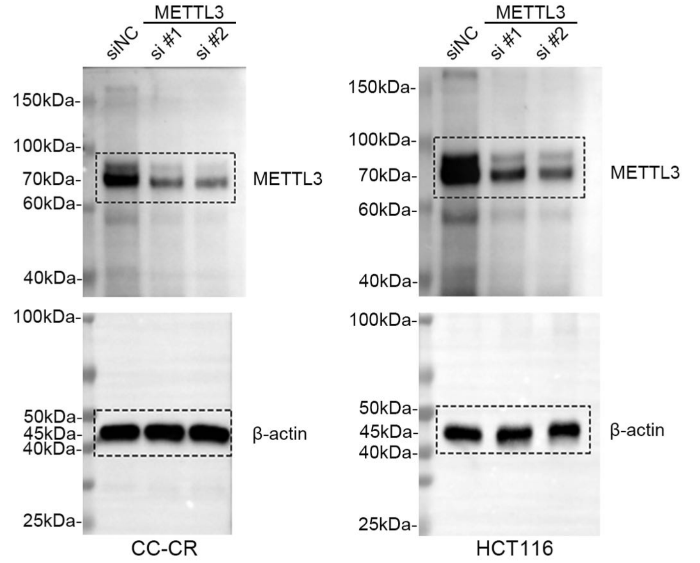

Figure 6b

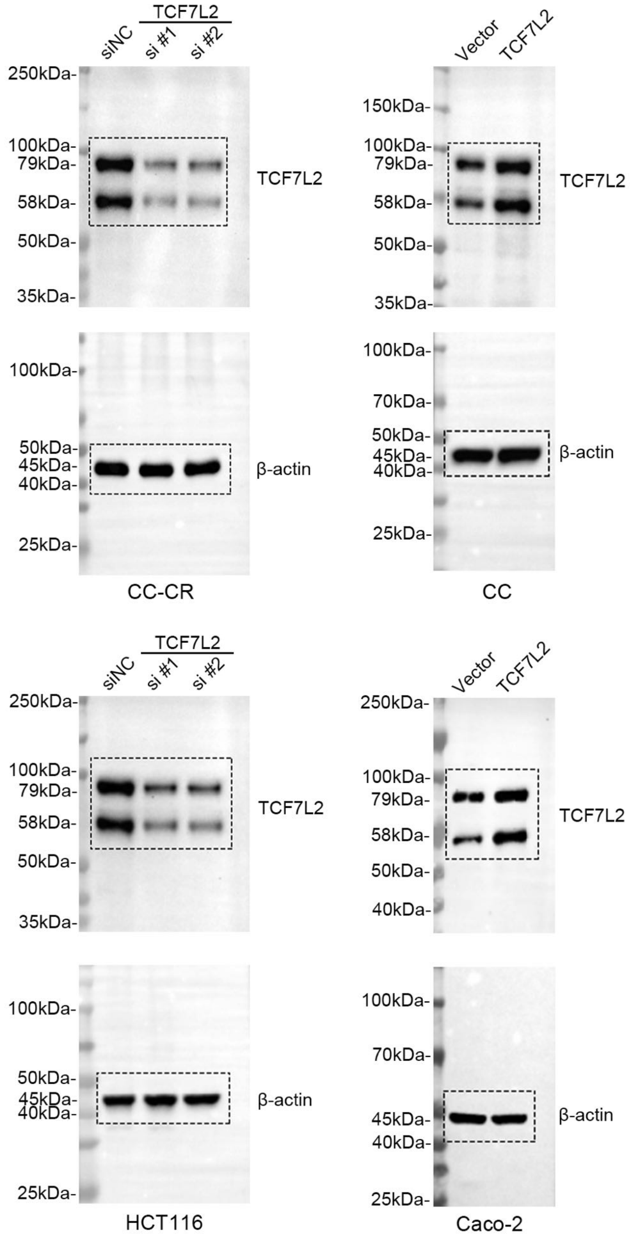

Figure 5g

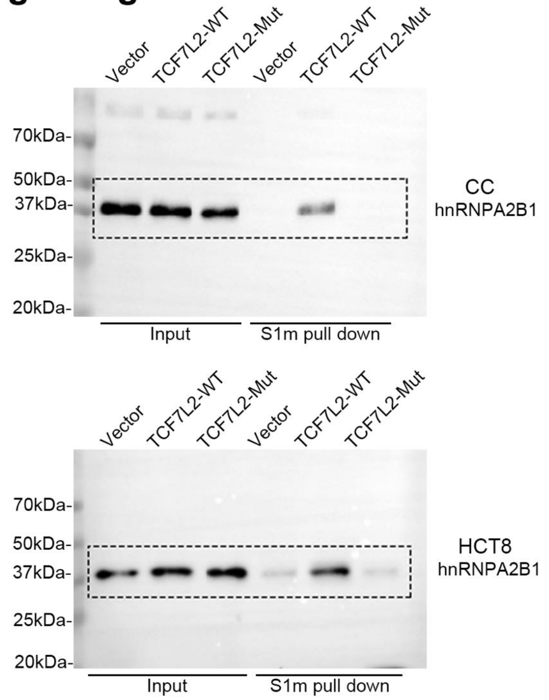

Figure S3a

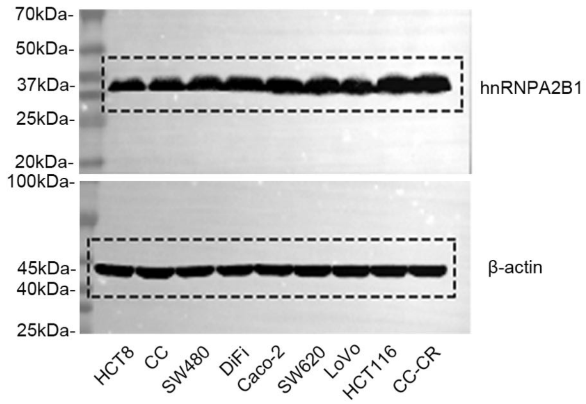

Figure S4c

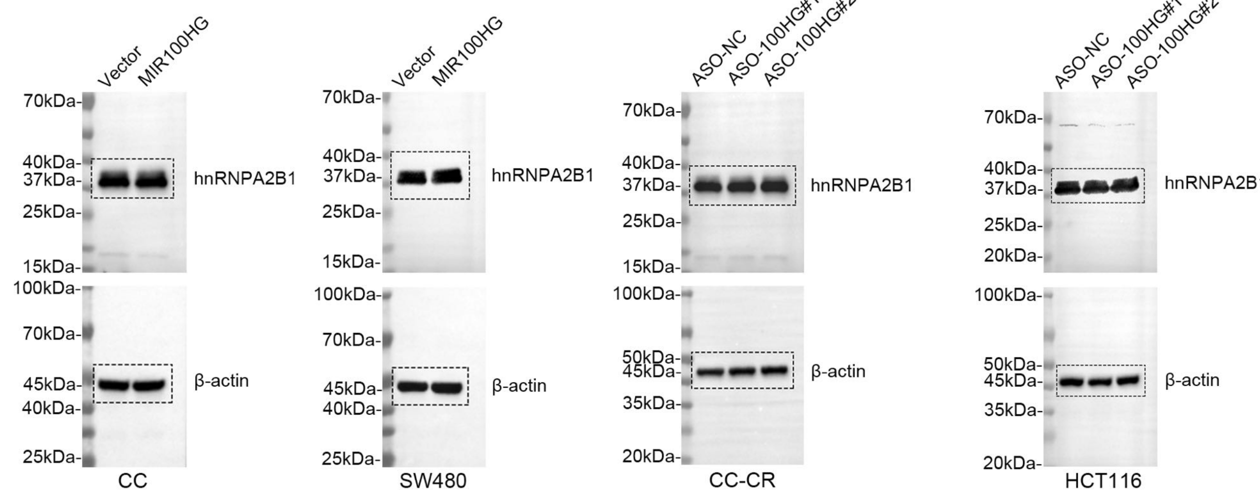

Figure S4d

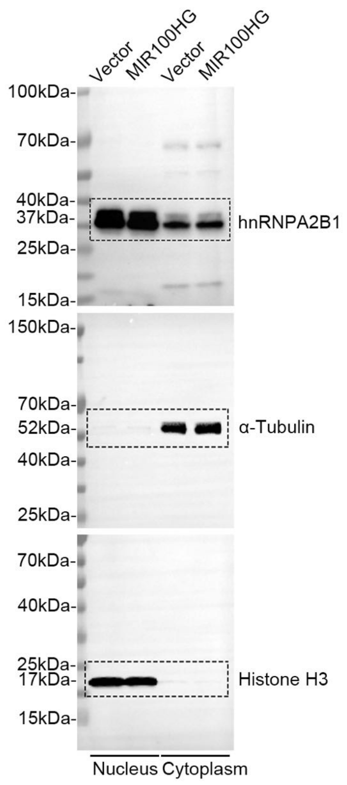

Figure S4e

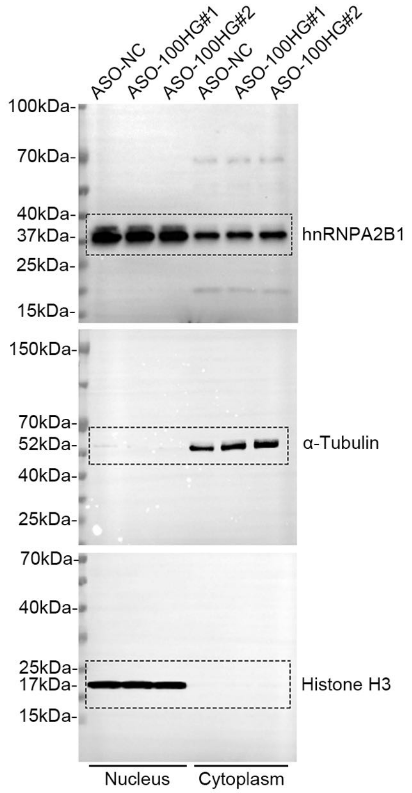

Figure S4f

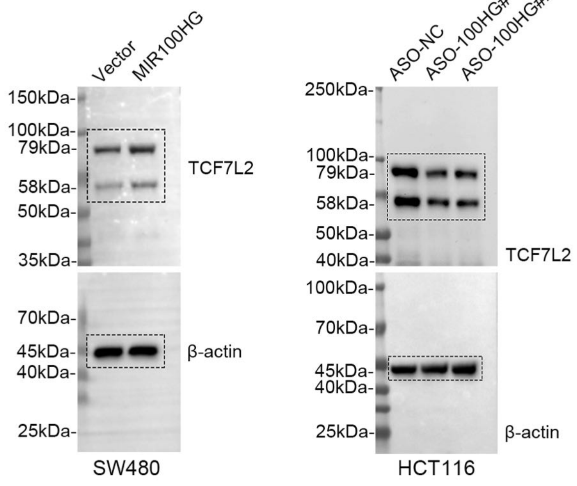

Figure S4g

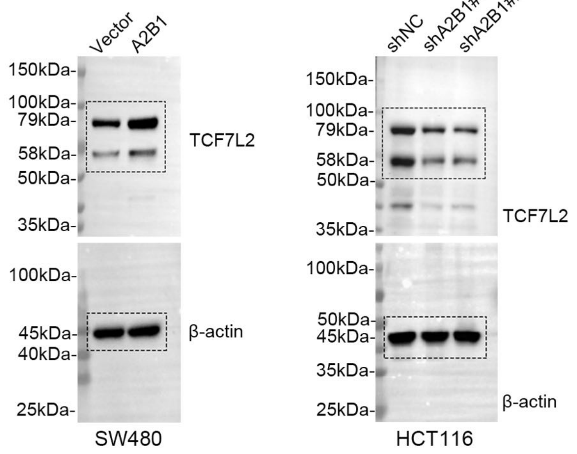

Figure S5b

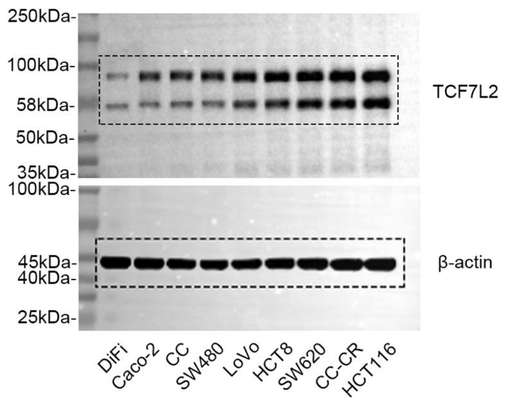

Supplement: Supplementary file 2 — Additional file 2. [file 12943_2022_1555_MOESM2_ESM.pdf]
